# Anticipating New Treatments for Cystic Fibrosis: A Global Survey of Researchers

**DOI:** 10.3390/jcm11051283

**Published:** 2022-02-26

**Authors:** Bernardo Cabral, Vito Terlizzi, Onofrio Laselva, Carlos Conte Filho, Fabio Mota

**Affiliations:** 1 Department of Economics, Federal University of Bahia, Salvador 40060-300, Brazil; bernardo.cabral@fiocruz.br; 2 Cystic Fibrosis Regional Reference Center, Department of Paediatric Medicine, Anna Meyer Children’s University, 50139 Florence, Italy; vito.terlizzi@meyer.it; 3 Department of Medical and Surgical Sciences, University of Foggia, 71122 Foggia, Italy; onofrio.laselva@unifg.it; 4 Department of Economics, Federal University of Santa Maria, Santa Maria 97105-900, Brazil; carlos.conte@fiocruz.br; 5 Laboratory of Cellular Communication, Oswaldo Cruz Institute, Oswaldo Cruz Foundation, Rio de Janeiro 21040-360, Brazil

**Keywords:** cystic fibrosis, CFTR modulator therapies, genetic therapies, survey, expert opinion

## Abstract

Cystic fibrosis is a life-threatening disease that affects at least 100,000 people worldwide. It is caused by a defect in the cystic fibrosis transmembrane regulator (CFTR) gene and presently, 360 CFTR-causing mutations have been identified. Since the discovery of the CFTR gene, the expectation of developing treatments that can substantially increase the quality of life or even cure cystic fibrosis patients is growing. Yet, it is still uncertain today which developing treatments will be successful against cystic fibrosis. This study addresses this gap by assessing the opinions of over 524 cystic fibrosis researchers who participated in a global web-based survey. For most respondents, CFTR modulator therapies are the most likely to succeed in treating cystic fibrosis in the next 15 years, especially through the use of CFTR modulator combinations. Most respondents also believe that fixing or replacing the CFTR gene will lead to a cure for cystic fibrosis within 15 years, with CRISPR-Cas9 being the most likely genetic tool for this purpose.

## 1. Introduction

Cystic fibrosis (CF) is the most common life-threatening autosomal recessive disease in Caucasian populations, and it is caused by mutations in the cystic fibrosis transmembrane conductance regulator (CFTR) gene. CF affects at least 100,000 people worldwide, half of them in Europe [1]. The CF phenotype is characterized by lung disease (bronchiectasis with persistent airway-based infection and inflammation), exocrine pancreatic insufficiency associated with nutrient malabsorption contributing to undernutrition, impaired growth, hepatobiliary manifestations, and male infertility [2,3]. Although historically considered a pediatric disease, over time, there have been substantial improvements in the survival of people with CF. Between 2015 and 2019, the average North American and European age of death for CF patients was 46.2 years [4] and 31.2 [1], respectively and is expected to increase in the coming years [5].

The CFTR protein is an ion channel that mediates chloride and bicarbonate transport in the epithelial cells of multiple organs, including the lungs, pancreas, and intestine [6]. To date, 360 CFTR-causing mutations have been identified (CFTR2: https://cftr2.org/, accessed on 7 October 2021) and classified into six distinct groups according to the type of defect in the CFTR gene: class I: protein synthesis defect; class II: maturation defect; class III: gating defect; class IV: conductance defect; class V: reduced quantity; and class VI: reduced stability [7]. The most common mutation that causes CF, c.1521_1523delCTT produces a protein lacking phenylalanine at position 508 (F508del, class II). This mutant CFTR protein exhibits defective processing, mistrafficking, and reduced functional expression as an anion channel at the cell surface, leading to abnormal fluid transport and impaired mucociliary clearance of bacteria—hallmark features of CF lung disease. However, pulmonary symptoms are the main cause of reduced quality of life and life expectancy, since they may lead to respiratory insufficiency [8,9,10].

The first US Food and Drug Administration (FDA) approved therapy for people who are homozygous for the F508del mutation was Orkambi^®^, in September 2016 (FDA: https://www.accessdata.fda.gov/drugsatfda_docs/nda/2018/211358Orig1s000TOC.cfm, accessed on 10 January 2021). It consists of a combination of lumacaftor (VX-809), a small molecule corrector that improves the F508del-mediated defects in protein processing and trafficking, and ivacaftor (VX-770), a potentiator that is effective in enhancing CFTR’s regulated channel activity at the cell surface [11]. More recently, the related Tezacaftor (or VX-661)-Ivacaftor combination has been approved as Symdeko^®^ [11,12], in June 2019 (FDA: https://www.fda.gov/news-events/press-announcements/fda-expands-approval-treatment-cystic-fibrosis-include-patients-ages-6-and-older, accessed on 10 January 2021). However, improvements in lung function in response to the Orkambi^®^ and Symdeko^®^ treatment are associated with modest clinical responsiveness. Therefore, in October 2019 (FDA: https://www.fda.gov/news-events/press-announcements/fda-approves-new-breakthrough-therapy-cystic-fibrosis, accessed on 10 January 2021), the FDA approved Trikafta^®^, the triple combination of two correctors, Elexacaftor (VX-445) and Tezacaftor, together with the potentiator, Ivacaftor, for patients bearing the F508del mutation in at least on one allele [13,14,15]. The potentiator Ivacaftor (Kalydeco^®^) was the first CFTR modulator and initiated the dynamic development of modulator therapy, focusing on treating patients with gating mutations [15]. It was approved by the FDA in January 2012 (FDA: https://www.accessdata.fda.gov/drugsatfda_docs/nda/2012/203188s000TOC.cfm, accessed on 10 January 2021).

Among these CFTR correctors and potentiators, a novel type of CFTR modulator was identified as an “amplifier”. This novel compound stabilizes CFTR mRNA and increases the total amount produced of the CFTR protein. Therefore, the amplifiers increase the protein abundance to be rescued by the CFTR corrector and potentiator [16,17]. Another type of CFTR modulator is called a “stabilizer”, which stabilizes the rescued CFTR protein at the plasma membrane and reduces its internalization rate [18,19].

An alternative to the CFTR modulators approach is to correct directly the genetic defect. Since the early 2010s, new researches with genetic therapies have shown potential to treat CF [20]. These findings include gene therapy (e.g., by delivering DNA or mRNA) and gene editing techniques (e.g., CRISPR/Cas9 approach) [21]. Gene therapy is “a technique that modifies a person’s genes to treat or cure disease” (FDA: fda.gov/vaccines-blood-biologics/cellular-gene-therapy-products/what-gene-therapy, accessed on 10 January 2021). In the case of CF, it includes a variety of therapeutic strategies that can place a new, correct version of the CFTR gene into the cells of CF patients by an adeno-associated virus, lentivirus, or liposomes vectors. Although the defective copies of the CFTR gene remain present, delivering the correct copy of the gene (Wt-CFTR) would give the cells the ability to make Wt-CFTR functional proteins [5,22,23]. On the other hand, gene-editing techniques use the cell’s DNA repair structures to correct the mutation in the DNA. Gene editing corrects mutations in the CFTR gene, which may allow the cure of individuals with CF, regardless of their mutation type [22,24].

Since the discovery of the CFTR gene in 1989 [25], the expectation of developing treatments capable of substantially increasing the quality of life or even curing CF patients is growing. However, is still an open question in regard to which treatments currently under development will be successful in fighting CF in the future. Our study addresses this issue by assessing the opinions of 524 CF-related researchers from around the world about the future of CF treatment. To do so, we carried out a global web-based survey of researchers, which are authors of recent scientific publications related to CF indexed in the Web of Science Core Collection (WoS).

As far as we know, just a few studies have tried to anticipate which the treatments have the greatest potential to deal with this disease. Some of them have looked at a specific drug line [26,27,28,29,30] and others have tried to visualize possible future alternatives through literature reviews [8,10,31]. However, none of these studies offers a long-term perspective of the future of CF treatment based on the views of a large number of researchers invested in it. Here, based on a literature review and looking 15 years ahead, we explore some promising therapeutic options and technologies that are leading to new treatments for CF that may perhaps provide a cure for the disease in the future. The goal of the paper is to anticipate technologies that might be relevant for treating and preventing CF through qualified information generation in order to inform relevant stakeholders, be they researchers or policy makers.

## 2. Materials and Methods

### 2.1. Literature Review and Questionnaire Design

We conducted a literature review aimed to identify relevant issues about the future of CF treatment. For this, we used editorials and review articles related to CF recently published in journals indexed in WoS, found according to the following search strategy:

TS = (“Cystic Fibrosis *” OR Mucoviscidosis OR “Fibrocystic Disease of Pancreas” OR “Pancreas Fibrocystic Disease *”) and TS = (future * or foresight * or forthcoming * or prospective * or imminent *) AND LANGUAGE: (English) AND DOCUMENT TYPES: (Editorial Material OR Review). Indexes = SCI-EXPANDED Timespan = 2017-jan/2021.

The search strategy used terms from the Medical Subject Headings (MeSH) of the U.S. National Library of Medicine (NCBI: ncbi.nlm.nih.gov/mesh, accessed on 10 January 2021) in combination with future-related terms selected freely. We used the WoS advanced search mode with the tag Topic (TS), which searches the title, abstract, and keywords of publications. Only the Science Citation Index Expanded (SCI-EXPANDED) was used to retrieve editorials and review articles published in scientific journals. We restricted the search period from 2017 to January 2021 to obtain recent publications on CF.

The search conducted in January 2021 retrieved 346 records of publications, which were imported as plain text into the data/text mining software VantagePoint 11.0. After reading their titles and abstracts, we preselected 47 records of publications. Then, we generated a list of their Digital Object Identifiers (DOIs) and imported it into the reference management software Citavi 6.1, where these references were downloaded in a PDF format and fully read. After this process, we considered 47 [6,8,9,10,20,22,23,24,26,27,28,29,30,31,32,33,34,35,36,37,38,39,40,41,42,43,44,45,46,47,48,49,50,51,52,53,54,55,56,57,58,59,60,61,62,63,64] references appropriate to the study and used them in the literature review that allowed us to draft the survey questionnaire.

The questionnaire considered a 15-year future horizon (2021–2036) and was divided into six parts. The first part is a survey introduction, informing respondents about the purpose of the study, aspects of data collection and treatment, anonymity, voluntary participation, and request for informed consent. The second part is a filter of the respondent’s level of knowledge about CF. Those who self-reported high, good, or some knowledge were qualified for the survey, while those declaring no knowledge were disqualified and did not answer the questionnaire. The third part consists of a question about which therapeutic option would be most likely to be successful in treating CF: (i) Genetic therapies, (ii) CFTR modulator therapies, or (iii) Other. Those respondents who selected genetic therapies or CFTR modulator therapies were then directed to a linked question related to the selected therapeutic option. In this question, respondents were asked to rank, from most likely to least likely, specific therapies related to the therapeutic option they pointed to as most promising. From the ranking assigned by each respondent, we calculate the weighted average of the responses by assigning weights to the response options. For CFTR modulators, with four therapeutic options, the option ranked first had weight 4, the second weight 3, the third weight 2, and the last weight 1. For genetic therapies, with five therapeutic options, the option ranked first had weight 5 and the last had weight 1. Thus, the higher the value of the weighted average, the higher the average expectation that a given therapy is promising in treating CF.

From the fourth part, all qualified respondents who agreed to participate in the survey answered the same questions. The fourth part asked about the likelihood of fixing or replacing the CFTR gene leading to a cure for CF. Those who indicated that a cure would be likely before or after 15 years were directed to a linked question, where they were to indicate, via a ranking, the approaches most likely to be successful in fixing or replacing the malfunctioning CFTR gene. With four gene editing approaches to be ranked, we calculated the weighted average of the respondents’ answers by assigning weight 4 to the option ranked first and weight 1 to the one ranked last. The fifth and sixth parts were optional, so the data collected were not included in the calculation of the number of completed questionnaires obtained. The fifth part is composed of five demographic questions (degree of education, years of experience, work institution, professional occupation, and geographical region of residence). The last part offered respondents a space to share any comments, criticisms, and suggestions.

### 2.2. Collecting Respondents in Scientific Publications

The respondents of this survey were identified in recent articles or review articles related to CF, published in journals indexed in WoS. To this end, the following search strategy was used:

TS = (“Cystic Fibrosis *” OR Mucoviscidosis OR “Fibrocystic Disease of Pancreas” OR “Pancreas Fibrocystic Disease *”) AND DOCUMENT TYPES: (Article OR Review) Indexes = SCI-EXPANDED Timespan = 2017-mar/2021.

In the WoS advanced search field, we used the tag TS and the same CF descriptors obtained in MeSH. We restricted the search period from 2017 to March 2021 and the document types to articles or review articles. We used only the SCI-EXPANDED to identify researchers who have published recent articles in peer-reviewed scientific journals.

The search conducted in March 2021 retrieved 9031 records of publications. These records were imported into VantagePoint 11.0, where 9026 author emails were retrieved. We then created a CSV file with data from these authors (email, name, and publication title). This file was then imported into a Python-based email linking software, which allowed linking 8332 emails (92.26% of the total) to their owners. In this way, we were able to forward personalized e-mails to the vast majority of respondents (i.e., with the names of the recipients and the titles of the articles that led them to be invited to the study).

### 2.3. Survey Management, Questionnaire Application, and Ethical Aspects

The list of 9026 emails of respondents was imported into the SurveyMonkey online survey platform, where the questionnaire was designed, and the survey was conducted. After the import, the number of emails dropped to 7745 due to the presence of 601 bounced emails and 226 emails from respondents who opted out of previous surveys conducted via SurveyMonkey.

Before the formal study, the questionnaire was validated through a pilot study with a random sample of 451 researchers (5% of the total number of emails). This phase allowed us to evaluate the questionnaire (application routine, internal logic, completion rate, time to complete, question-wording, etc.) and to collect feedback from respondents. The questionnaire was available for completion for eight consecutive days after the invitation email was sent. During this period, up to three reminder emails were sent to non-respondents. The 18 researchers who participated in the pilot study made no comments that would indicate the need for changes to the questionnaire. Thus, the questionnaire and its application routine were not modified, and the data collected in the pilot were included in the analysis of the survey results.

The pilot and formal study were conducted in April and May 2021. Before completing the questionnaire, respondents were informed that the study was for research purposes only, that sensitive data would not be collected, and that their responses would be anonymized in the results. Respondents were made aware that informed consent would be given by answering the questionnaire. Thus, all researchers who answered the questionnaire gave their consent for use, for research purposes, and of any data collected in the survey. Given the voluntary participation, absence of sensitive issues, and anonymity of responses, it was not necessary to consult a research ethics committee.

### 2.4. Statistical Analysis

In parametric tests, the values of the studied variables must have a normal distribution or a normal approximation. Nonparametric tests, on the other hand, do not require knowledge of the distribution of the variable in the population. In addition, they can be applied to small samples. Having this premise as a starting point, we analyzed the distribution of the sample to verify if it has a normal distribution. The sample consists of questionnaires answered by respondents with high or good knowledge. Although respondents with some knowledge about CF qualified for the survey and answered the questionnaire, we chose to analyze only the responses from the two groups of respondents with the highest level of knowledge. The responses of some knowledge respondents are available in the Supplementary Materials.

We apply two tests to analyze the distribution of the sample: The Shapiro–Wilk test and the Kolmogorov–Smirnov. The Shapiro–Wilk test was originally restricted to a sample with a size of fewer than 50 observations. This test has become one of the preferred ones due to its good power properties. That is, no prior knowledge about the data distribution (mean and variance) is needed. The Kolmogorov–Smirnov test is an adherence distribution test particularly suitable for continuous distributions with a high number of observations. It can be applied to analyze whether a distribution is of normal, uniform, exponential, or Poisson type. When this test is used to verify the normal distribution of the sample, the Lillefors correction should be applied because without it the test tends to accept the null hypothesis.

The results of the Shapiro–Wilk and Kolmogorov–Smirnov tests showed that the sample does not follow a normal distribution (Supplementary Materials). Thus, we used three non-parametric tests to analyze the data collected in the survey: the binomial test, the Mann–Whitney U test, and the Wilcoxon rank test.

The binomial test was used to assess whether good knowledge and high knowledge respondents are statistically homogeneous or if there is a preponderance of one group over the other. The binomial test is applied to populations whose elements have two attributes of interest (dichotomous data). The population is assumed to be a *p* parameter Bernoulli from which a sample of size n is taken. Therefore, this test is used to test the *p* proportion of one of the attributes of the sample under analysis. Thus, it was analyzed whether the sample follows a distribution of 50% for each level of knowledge (high and good knowledge).

We applied the Mann–Whitney U test to assess whether the level of knowledge of the respondents interferes with the responses obtained. This test ranks the values contained in the sample and divides them among interest groups to analyze whether they are homogeneous or not [65]. When the results of the Mann–Whitney U test indicated that the level of knowledge interfered with the answers obtained, we analyzed frequency distributions to identify where the difference occurred. Following the Mann–Whitney U test results, this complementary analysis was performed on the two ranking questions of the questionnaire.

Finally, the Wilcoxon rank test was used to assess the standard response of the respondents. This test ranks the respondents’ answers in ascending order, assigning value 1 to the lowest answer position, value 2 to the second position, and so on. From this classification, we analyzed the median of the sample to assess whether there was a statistically a significant pattern of responses [66]. All data analysis was performed using IBM-SPSS Statistics 26. The complete results of the statistical analysis are available in the Supplementary Materials.

### 2.5. Limitations of the Study

The methods we applied to find, collect, and survey the respondents are based on previous future studies [67,68,69,70,71,72]. Similarly to other studies, this study has some limitations worth mentioning. First, collecting respondents in articles limits their variability. For the most part, they are restricted to researchers who are experts in a particular subject. While variability is desirable, they are nevertheless the most qualified to report on the future of emerging technologies, especially when uncertainty about the future is high. Second, as they are invested in the technologies they help develop, their expectations about the future may be susceptible to bias, especially optimism, either about the outcome or the time in which it will occur. The possibility of bias in experts’ opinions is well known in survey research and has not prevented the use of researchers as a reliable source of knowledge when the aim is to anticipate future possibilities, changes, trends, etc., that may be opened by emerging technologies. Third, it is not possible to assess the respondents’ knowledge of the subject. In the questionnaire, the level of knowledge is indicated by the respondents themselves. In any case, since all of them are authors of articles related to the survey’s subject published in peer-reviewed journals, the chances of collecting the opinion of non-knowledgeable respondents are low. Fourth, on the one hand, the time frame of the survey (15 years into the future) may perhaps be considered too long for expectations about the future to be reliable. On the other hand, this is too short a time frame to translate the new therapies from bench to bedside. To overcome such limitations and improve the reliability of expectations, some studies have assessed changes in experts’ expectations over time [69].

## 3. Results

**Figure 1:**
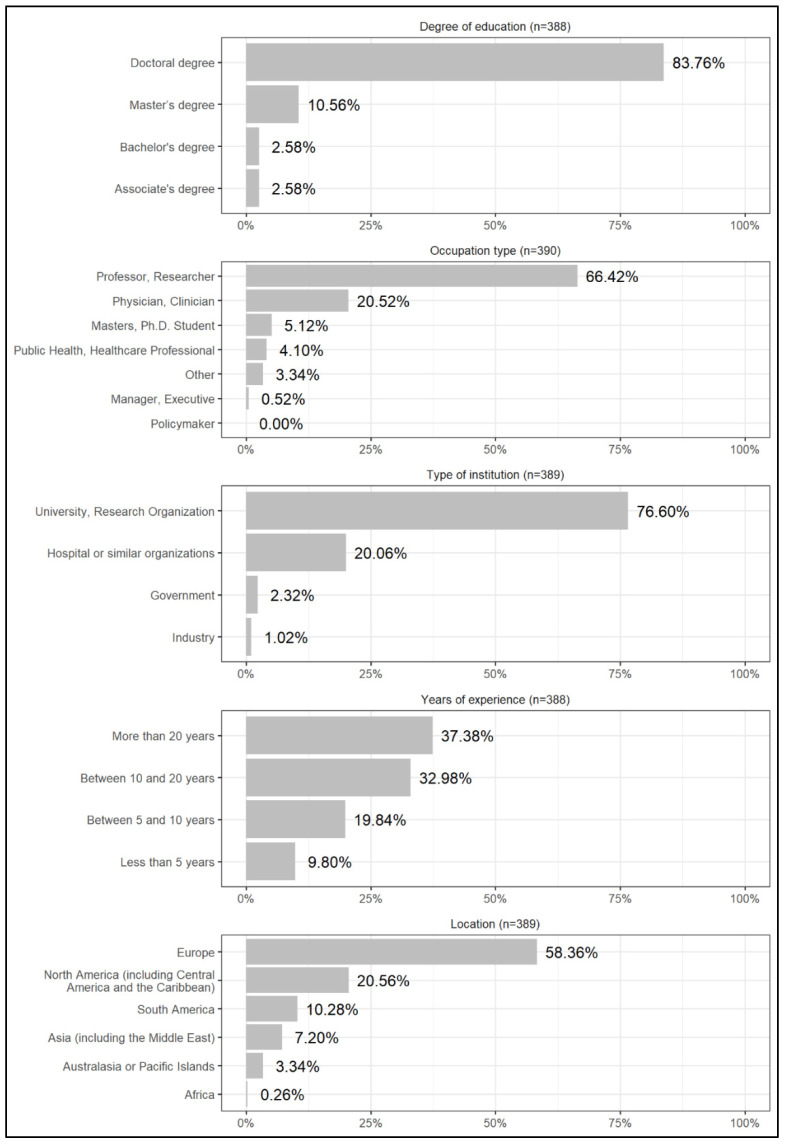
Demographic profile of the respondents.

Of the 7745 invited researchers, 524 accepted to take part in the study, giving us a response rate of 6.76%. Among these, 179 (34.16%), 223 (42.56%), and 114 (21.76%) reported having high, good, and some knowledge of CF, respectively. The 1.53% (8 respondents) that reported having no knowledge of the subject were disqualified from the survey, not answering the questionnaire. As mentioned in the Methods section, although qualified for the survey, the answers of some knowledge respondents are not included in the results as we chose to report the opinions of the two groups of researchers with greater knowledge on the topic. The answers of some knowledge respondents are available in the Supplementary Material. Of the 401 questionnaires of high and good knowledge respondents, 391 were completely filled (97.5% of total valid responses). Considering only the fully completed questionnaires, we obtained a representative sample with a 95% confidence level and a margin of error of 4.8%. The demographics of the respondents are presented in Figure 1.

### 3.1. Statistical Analysis

The relevant results of the statistical analysis are shown in Table 1. The result of the binomial test indicates that high knowledge respondents are preponderant in the sample. The results of the Mann–Whitney U test suggest that the level of knowledge influences the answer about which therapeutic option is more likely to be successful in treating CF within the next 15 years. Analyzing the frequency distribution, it is observed that 76.97% of high knowledge and 67.71% of good knowledge respondents believe that CFTR modulator therapies are the most likely to succeed. At the same time, 16.29% and 5.06% of high knowledge respondents believe, respectively, that genetic therapies and other therapies will be the most successful. Among good knowledge respondents, 22.42% believe in genetic therapies and 6.28% in other therapies. That is, these results suggest that respondents with good knowledge are more prone to choose genetic therapies or other therapies over CFTR modulator therapies than respondents with high knowledge. Finally, considering all respondents of the sample, the Wilcoxon rank test showed that the CFTR modulator therapies are the predominant therapeutic option.

### 3.2. Descriptive Statistics

The 401 respondents with high and good knowledge were asked to indicate which therapeutic option would be, in their view, the most likely to be successful in treating CF in the next 15 years. The vast majority (73.85%) choose CFTR modulators therapies as the most likely. In turn, genetic therapies were chosen by 20.26% of them. The responses of the 5.90% of respondents who believe that neither of these two therapeutic options would succeed in treating CF are available in the Supplementary Material.

**Figure 2:**
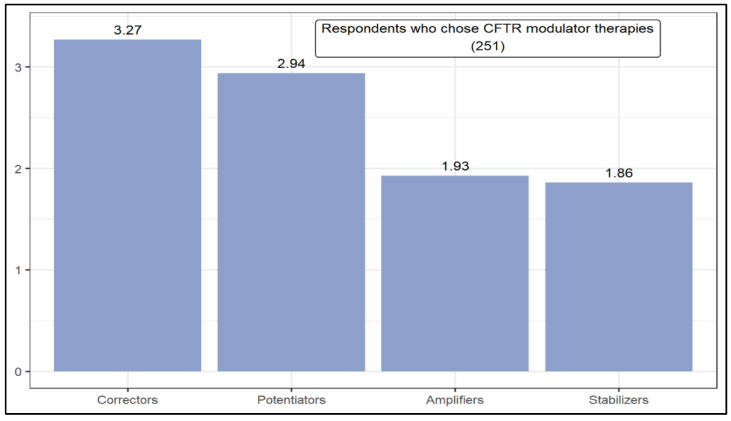
Ranking of cystic fibrosis transmembrane regulator (CFTR)modulator therapies.

**Figure 3:**
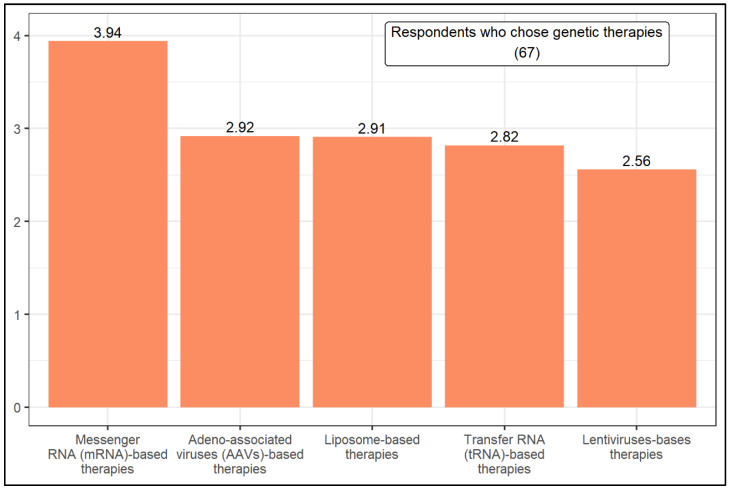
Ranking of genetic-based therapies.

**Figure 4:**
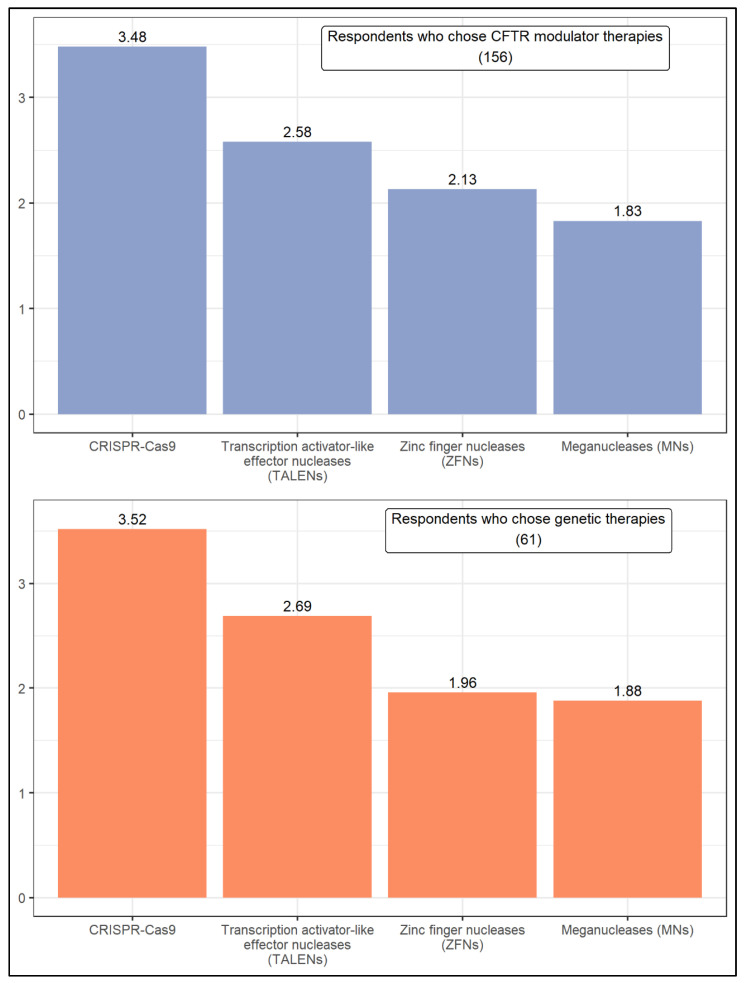
Ranking of approaches aimed at fixing or replacing the malfunctioning CFTR gene.

Figure 2 presents the ranking of modulators with the greatest therapeutic potential in CF, according to the opinion of respondents who chose CFTR modulator therapies. Correctors (3.27) and potentiators (2.94) had the highest weighted averages. The Y-axis for Figure 2, Figure 3 and Figure 4 are the weighted average of each option according to their position in the rankings. According to the Mann–Whitney U test, the level of respondents’ knowledge influences the likelihood of choosing a given modulator. In this case, compared to good knowledge, respondents with a high knowledge are more likely to choose correctors or potentiators over stabilizers.

In turn, the respondents who selected genetic therapies were asked to rank five genetic-based therapies according to the likelihood of being successful in treating CF in the next 15 years (Figure 3). They are Transfer RNA (tRNA)-based therapies, Adeno-associated viruses (AAVs)-based therapies, Messenger RNA (mRNA)-based therapies, Lentiviruses-based therapies, and Liposome-based therapies. With a weighted average of 3.94, mRNA-based therapies stand out from the others in respondents’ preference. The Mann–Whitney U test showed that the level of knowledge influences the likelihood of choosing a given genetic-based therapy. Compared to high knowledge, good knowledge respondents are more likely to choose AAVs-based therapies.

**Figure 5:**
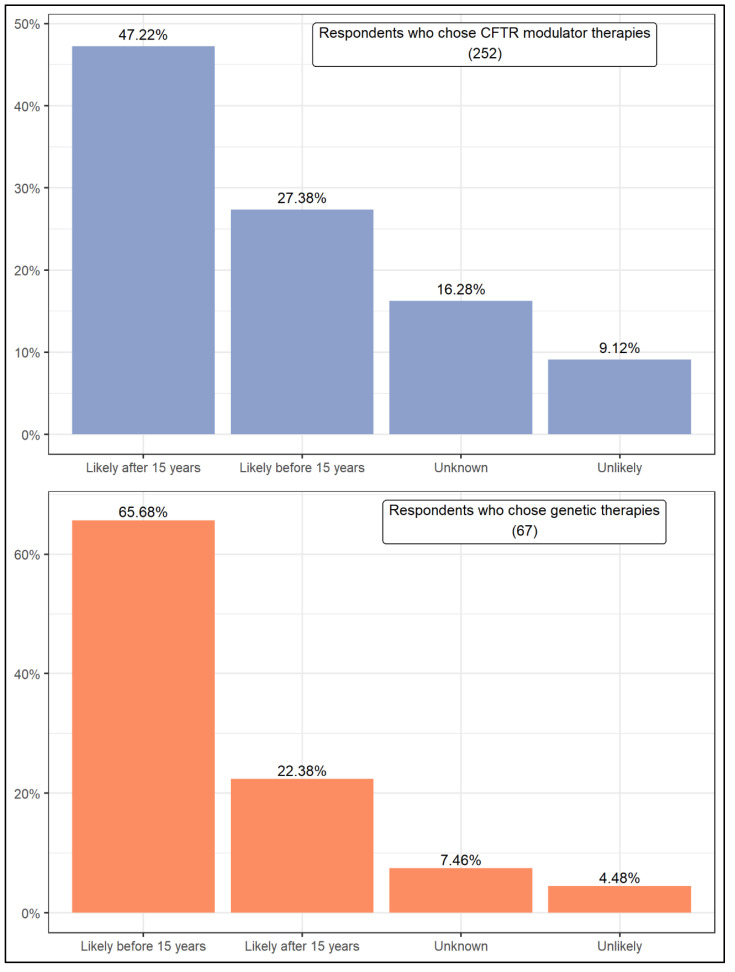
Expectation that fixing or replacing the CFTR gene will lead to a cure for CF.

Presently, all respondents answered the same questions. Figure 5 presents respondents’ expectations about the likelihood of a cure for CF by fixing or replacing the CFTR gene. Of those who chose CFTR modulator therapies, 47.22% believe that a cure is likely to occur after 15 years. Even so, taken together, the respondents who believe that a cure is likely total 74.60%. About 25% of them believe that a cure is unknown or unlikely. In turn, the respondents who chose genetic therapies seem to be more optimistic as about two-thirds of them (65.68%) believe that a cure is likely to occur before 15 years. Another 22.38% believe this outcome is likely after 15 years, comprising that more than 90% of the respondents expect a cure to this disease to occur in the future. Compared to the first group of respondents, a much smaller percentage (11.94%) indicated that a cure for CF is unlikely or unknown. In addition, the Wilcoxon rank test results are the opposite for the two groups of respondents. While respondents who chose CFTR modulator therapies expect a cure to take more than 15 years, those who chose genetic therapies believe it will take less time.

All respondents that reported a cure would be likely were asked to rank four approaches according to the likelihood of being successful in fixing or replacing the malfunctioning CFTR gene in the next 15 years (Figure 5). They are zinc finger nucleases (ZFNs), transcription activator-like effector nucleases (TALENs), meganucleases (MNs), and clustered regularly interspaced short palindromic repeats and CRISPR-associated protein 9 (CRISPR-Cas9). A similar pattern is observed in the responses of respondents who chose CFTR modulator therapies and genetic therapies. With a weighted average of about 3.5, CRISPR-Cas9 was by far the approach considered most promising by both groups of respondents, followed by TALENs (with a weighted average ranging from 2.58 to 2.69).

## 4. Discussion

Today new drugs that target the basic defect in CF have provided hope for patients and progress in drug development over the past decade [73,74,75]. CFTR modulator drugs restore the CFTR function, and this is associated with an amazing improvement in the respiratory function and nutritional status, and enhanced quality of life in patients carrying the F508del mutation as well as those with mutations that affect gating, conductance, or protein folding. Although the F508del is the most prevalent CF-causing mutation, the frequency of allele occurrence in CF varies greatly among racial and ethnic groups. Furthermore, CF patients with the same mutations often display high interpatient variability in disease phenotypes and responses to CFTR modulator therapies due to differences in the modifier gene status and genetic backgrounds [74].

Gene therapy and gene editing are regarded as the most promising new therapeutical approaches to cope with CFTR mutations from all six classes. Our results show that, despite both approaches still being in a pre-clinical development phase, most respondents appeared optimistic about the potential of this new drugs to reduce the CF burden in coming years.

Additionally, with a weighted average of 3.94, CF researchers chose mRNA-based therapies as the most promising therapeutic strategy for CF treatment (Figure 3). The mRNA therapy via nanoparticle delivery represents a powerful technology for transferring genetic material to cells, including airway epithelial cells. Recently, it was demonstrated that lipid-based nanoparticles with chemically modified CFTR mRNA rescued the functional expression of CFTR in a CF airway epithelial cell line and nasal epithelial cells from CFTR knockout mice [75].

Gene therapy uses both viral and non-viral (i.e., liposomes) vectors to deliver the nucleic acid to the cells. Previous studies investigated the use of AAV or lentivirus to restore the CFTR function in CF airway epithelial cells and CF animal models including pigs, sheep, ferrets, and mice [29,76,77,78,79,80,81,82,83,84]. In this study, the CF researchers that ranked genetic therapies as a promising approach evenly chose genetic therapies based on AAVs (2.92), liposome (2.91), tRNA (2.82), and lentivirus (2.56). Although gene therapy carries promise, it has several limitations. One of the main limitations is their delivery methods. This may cause mucus, versatile immune responses, and considerably impaired gene transfer into the lung [85]. Therefore, the selection of a delivery method is important. However, our results do not show a clear idea of which vector will hold the most promise in the future (Figure 3).

We also asked the two groups of CF researchers which approach they would choose to fix the CFTR gene using gene editing. Of the given options, both groups of CF researchers who chose CFTR modulators and genetic therapies as the promising CF treating method, considered the CRISPR-Cas9 as the most promising therapy to fix the CFTR gene. Recently, it has also been demonstrated that by using the CRISPR-Cas9, it is possible to correct the CFTR mutation in airway epithelial cells and patient-derived organoids, independently to which mutation the CF patient is bearing [86,87,88,89,90,91]. Following CRISPR-Cas9, both groups of respondents chose TALENs, which recently has been demonstrated to be effective in repairing the F508del mutation in iPSC-derived intestinal organoids [92]. Lastly, both groups showed less optimism for gene editing by ZFNs and MNs. These ideas may coincide as no studies demonstrate that MNs could be used to repair the CFTR gene while only two studies have demonstrated the functional rescue of F508del-CFTR by ZFN homology-directed repair [93,94].

## 5. Conclusions

In this paper, we surveyed CF-related researchers around the world on their opinion of the future of CF treatment within the next 15 years. Our results showed that the level of knowledge influences these answers: most respondents with high or good knowledge believe that CFTR modulator therapies are the most likely to succeed. On the other hand, a non-negligible percentage of respondents with good knowledge (16.29% and 5.06%) revealed to be more prone to choosing genetic therapies or other therapies. However, CFTR modulator therapies are the predominant therapeutic option. Correctors and potentiators had the highest weighted averages, compared to amplifiers and stabilizers, and respondents with high knowledge chose them more frequently. As for gene editing, most respondents believe it will play an important role in the search of a cure for CF in the next 15 years, particularly using CRISPR-Cas9.

## Figures and Tables

**Table 1:** Summary of the statistical analysis of the sample.

Test		Results
Normality test	Shapiro–Wilk	The sample did not show normal distribution.
	Kolmogorov–Smirnov	The sample did not show normal distribution.
Binomial test		The sample was not homogeneously distributed
Mann–Whitney U		The level of knowledge influences the result in the following situations: (1) Which therapeutic option is most likely to be successful in treating cystic fibrosis in the next 15 years. (2) CFTR modulator therapies: potentiators, correctors, and stabilizers. (3) Genetic therapies: Adeno-associated viruses-based therapies.
Wilcoxon rank test		(1) CFTR modulator therapies are the predominant option. (2) Respondents who choose CFTR modulator therapies expect that a cure will take more than 15 years. (3) Respondents who choose genetic therapies expect that a cure will take less than 15 years.

## Supplementary Materials

The following supporting information can be downloaded at: https://www.mdpi.com/article/10.3390/jcm11051283/s1, File S1: **SUPPLEMENTARY MATERIAL**.
