# Supplementary material for: Anticipating New Treatments for Cystic Fibrosis: A Global Survey of Researchers
Source: J Clin Med. 2022 Feb 26;11(5):1283. doi: 10.3390/jcm11051283 (PMC8911007; doi:10.3390/jcm11051283)

# SUPPLEMENTARY MATERIAL

1 Statistical Analysis of the survey ........ 2

1.1 Please indicate your knowledge level on cystic fibrosis ........ 2

1.2 Which of the following therapeutic options is most likely to be successful in treating cystic fibrosis in the next 15 years? ........ 2

1.3 Respondents who selected CFTR modulator therapies as the most likely to be successful in treating CF in the next 15 years ........ 3

1.4 Respondents who selected genetic therapies as the most likely to be successful in treating CF in the next 15 years ........ 6

1.5 Respondents who selected 'other' option as the most likely to be successful in treating CF in the next 15 years ........ 9

2 Descriptive statistics of the results according to the chosen therapeutic option and the respondents' level of knowledge ........ 11

2.1 Respondents who selected CFTR modulator therapies as the most likely to be successful in treating CF in the next 15 years ........ 11

2.2 Respondents who selected genetic therapies as the most likely to be successful in treating CF in the next 15 years ........ 16

2.3 Respondents who selected 'other' option as the most likely to be successful in treating CF in the next 15 years ........ 22

# 1 Statistical Analysis of the survey

## 1.1 Please indicate your knowledge level on cystic fibrosis

| | | | |
|---|---|---|---|
| **Sample size** | | 401 | |
| **Margin of error** | | 4.8% | |
| **Mean** | | 2.48 | |
| **Standard deviation** | | 0.852 | |
| **Percentiles** | ¼ | 2 (good knowledge) | |
| | ½ | 2 (good knowledge) | |
| | ¾ | 3 (high knowledge) | |
| **Binomial test** | | **Test ratio** | **P-value** |
| | | 0.50 | 0.028 |
| **Kolmogorov-Smirnov** | | **Test ratio** | **P-value** |
| | | 0.370 | 0.000 |
| **Shapiro-Wilk** | | **Test ratio** | **P-value** |
| | | 0.632 | 0.000 |

## 1.2 Which of the following therapeutic options is most likely to be successful in treating cystic fibrosis in the next 15 years?

| Therapy | Number of responses |
|---|---|
| CFTR modulator Therapies | 288 |
| Genetic Therapies | 18 |
| Other Therapies | 95 |

| | | | |
|---|---|---|---|
| **Sample size** | | 401 | |
| **Margin of error** | | 4.8% | |
| **Mean** | | 3.38 | |
| **Standard deviation** | | 0.822 | |
| **Percentiles** | ¼ | 2 (Genetic therapies) | |
| | ½ | 3 (CFTR modulator therapies) | |
| | ¾ | 3 (CFTR modulator therapies) | |
| **Mann-Whitney U** | | **Statistic** | **P-value** |
| | | -1.996 | 0.046 |
| **Wilcoxon (H0: median = 3)** | | **Statistic** | **P-value** |
| | | -0.780 | 0.435 |
| **Kolmogorov-Smirnov** | | **Statistic** | **P-value** |
| | | 0.447 | 0.000 |
| **Shapiro-Wilk** | | **Statistic** | **P-value** |
| | | 0.577 | 0.000 |

| | | Other Therapies | Genetic Therapies | CFTR Modulator Therapies | Total |
|---|---|---|---|---|---|
| Good knowledge | Number of observations | 14 | 50 | 151 | 223 |
| | Frequency | 6.28% | 22.42% | 67.71% | 100.0% |

| | | | | | |
|---|---|---|---|---|---|
| High Knowledge | Number of observations | 9 | 29 | 137 | 178 |
| | Frequency | 5.06% | 16.29% | 76.97% | 100.0% |

## 1.3 Respondents who selected CFTR modulator therapies as the most likely to be successful in treating CF in the next 15 years

### 1.3.1 Please rank the following approaches from most likely to least likely to be successful in treating cystic fibrosis in the next 15 years

| | Most Likely (1) | 2 | 3 | Least likely (4) |
|---|---|---|---|---|
| Potentiators | 25.50% | 51.40% | 14.60% | 8.50% |
| Correctors | 57.00% | 23.30% | 9.20% | 10.40% |
| Amplifiers | 8.30% | 12.50% | 42.90% | 36.30% |
| Stabilizers | 9.10% | 12.40% | 34.00% | 44.40% |

| | | Potentiators | Correctors | Amplifiers | Stabilizers |
|---|---|---|---|---|---|
| Mann-Whitney U | Statistic | -2.080 | -2.114 | -0.223 | -3.840 |
| | P-value | 0.037 | 0.035 | 0.823 | 0.000 |

| Potentiators | | | | | | |
|---|---|---|---|---|---|---|
| | | 1 | 2 | 3 | 4 | Total |
| Good knowledge | Number of observations | 29 | 53 | 25 | 13 | 120 |
| | Frequency | 24.2% | 44.2% | 20.8% | 10.8% | 100.0% |
| High Knowledge | Number of observations | 34 | 74 | 11 | 8 | 127 |
| | Frequency | 26.8% | 58.3% | 8.7% | 6.3% | 100.0% |

| Correctors | | | | | | |
|---|---|---|---|---|---|---|
| | | 1 | 2 | 3 | 4 | Total |
| Good knowledge | Number of observations | 62 | 28 | 15 | 16 | 121 |
| | Frequency | 51.2% | 23.1% | 12.4% | 13.2% | 100.0% |
| High Knowledge | Number of observations | 80 | 30 | 8 | 10 | 128 |
| | Frequency | 62.5% | 23.4% | 6.3% | 7.8% | 100.0% |

| Amplifiers | | | | | | |
|---|---|---|---|---|---|---|
| | | 1 | 2 | 3 | 4 | Total |
| Good knowledge | Number of observations | 10 | 18 | 42 | 46 | 116 |
| | Frequency | 8.6% | 15.5% | 36.2% | 39.7% | 100.0% |
| High Knowledge | Number of observations | 10 | 12 | 61 | 41 | 124 |
| | Frequency | 8.1% | 9.7% | 49.2% | 33.1% | 100.0% |

| Stabilizers | | | | | | |
|---|---|---|---|---|---|---|
| | | 1 | 2 | 3 | 4 | Total |
| Good knowledge | Number of observations | 19 | 19 | 37 | 41 | 116 |
| | Frequency | 16.4% | 16.4% | 31.9% | 35.3% | 100.0% |
| High Knowledge | Number of observations | 3 | 11 | 45 | 66 | 125 |
| | Frequency | 2.4% | 8.8% | 36.0% | 52.8% | 100.0% |

### 1.3.2 Fixing or replacing the CFTR gene will lead to a cure for cystic fibrosis

| | | | |
|---|---|---|---|
| Sample size | | 252 | |
| Margin of error | | 6.1% | |
| Mean | | 2.86 | |
| Standard deviation | | 1.00 | |
| Percentiles | ¼ | 2 (Unlikely) | |
| | ½ | 3 (likely after 15 years) | |
| | ¾ | 4 (likely before 15 years) | |
| Mann-Whitney U | | Statistic | P-value |
| | | -0.429 | 0.668 |
| Wilcoxon (H0: median = 3) | | Statistic | P-value |
| | | 1.252 | 0.210 |
| Kolmogorov-Smirnov | | Statistic | P-value |
| | | 0.303 | 0.000 |
| Shapiro-Wilk | | Statistic | P-value |
| | | 0.812 | 0.000 |

| | | Unknown | Unlikely | Likely after 15 years | Likely before 15 years | Total |
|---|---|---|---|---|---|---|
| Good knowledge | Number of observations | 25 | 9 | 54 | 35 | 123 |
| | Frequency | 20.3% | 7.3% | 43.9% | 28.5% | 100.0% |
| High Knowledge | Number of observations | 16 | 14 | 65 | 34 | 129 |
| | Frequency | 12.4% | 10.9% | 50.4% | 26.4% | 100.0% |

### 1.3.3 Please rank the following approaches from most likely to least likely to be successful in fixing or replacing the malfunctioning CFTR gene in the next 15 years

| | Most likely (1) | 2 | 3 | Least likely (4) |
|---|---|---|---|---|
| CRISPR-Cas9 | 72.70% | 13.00% | 3.90% | 10.40% |
| TALENs | 14.80% | 43.60% | 26.80% | 14.80% |
| Meganucleases | 8.10% | 11.50% | 35.80% | 44.60% |
| Zinc Finger Nucleases (ZFNs) | 5.40% | 31.10% | 34.50% | 29.10% |

| | | CRISPR-Cas9 | TALENs | Meganucleases | Zinc Finger Nucleases (ZFNs) |
|---|---|---|---|---|---|
| Mann-Whitney U | Statistic | -1.775 | -1.431 | -.122 | -.342 |
| | P-value | 0.076 | 0.152 | 0.903 | 0.732 |

| CRISPR-Cas9 | | | | | | |
|---|---|---|---|---|---|---|
| | | 1 | 2 | 3 | 4 | Total |
| Good knowledge | Number of observations | 47 | 11 | 2 | 11 | 71 |
| | Frequency | 66.2% | 15.5% | 2.8% | 15.5% | 100.0% |
| High Knowledge | Number of observations | 65 | 9 | 4 | 5 | 83 |
| | Frequency | 78.3% | 10.8% | 4.8% | 6.0% | 100.0% |

| TALENs | | | | | | |
|---|---|---|---|---|---|---|
| | | 1 | 2 | 3 | 4 | Total |
| Good knowledge | Number of observations | 12 | 31 | 16 | 8 | 67 |
| | Frequency | 17.9% | 46.3% | 23.9% | 11.9% | 100.0% |
| High Knowledge | Number of observations | 10 | 34 | 24 | 14 | 82 |
| | Frequency | 12.2% | 41.5% | 29.3% | 17.1% | 100.0% |

| Meganucleases | | | | | | |
|---|---|---|---|---|---|---|
| | | 1 | 2 | 3 | 4 | Total |
| Good knowledge | Number of observations | 7 | 6 | 25 | 30 | 68 |
| | Frequency | 10.3% | 8.8% | 36.8% | 44.1% | 100.0% |
| High Knowledge | Number of observations | 5 | 11 | 28 | 36 | 80 |
| | Frequency | 6.3% | 13.8% | 35.0% | 45.0% | 100.0% |

| Zinc Finger Nucleases (ZFNs) | | | | | | |
|---|---|---|---|---|---|---|
| | | 1 | 2 | 3 | 4 | Total |
| Good knowledge | Number of observations | 5 | 19 | 25 | 18 | 67 |
| | Frequency | 7.5% | 28.4% | 37.3% | 26.9% | 100.0% |
| High Knowledge | Number of observations | 3 | 27 | 26 | 25 | 81 |
| | Frequency | 3.7% | 33.3% | 32.1% | 30.9% | 100.0% |

## 1.4 Respondents who selected genetic therapies as the most likely to be successful in treating CF in the next 15 years

### 1.4.1 Please rank the following therapies from most likely to least likely to be successful in treating CF in the next 15 years

| | Most likely (1) | 2 | 3 | 4 | Least likely (5) |
|---|---|---|---|---|---|
| Adeno-associated viruses (AAVs)-based therapies | 17.70% | 16.10% | 25.80% | 21.00% | 19.40% |
| Lentiviruses-based therapies | 9.40% | 17.20% | 15.60% | 35.90% | 21.90% |
| Liposome-based therapies | 19.70% | 21.20% | 19.70% | 9.10% | 30.30% |
| Messenger RNA (mRNA)-based therapies | 43.90% | 24.20% | 18.20% | 9.10% | 4.50% |
| Transfer RNA (tRNA)-based therapies | 12.30% | 23.10% | 20.00% | 23.10% | 21.50% |

| | | Adeno-associated viruses (AAVs)-based therapies | Lentiviruses-based therapies | Liposome-based therapies | Messenger RNA (mRNA)-based therapies | Transfer RNA (tRNA)-based therapies |
|---|---|---|---|---|---|---|
| Mann-Whitney U | Statistic | -2.120 | -0.232 | -1.067 | -0.055 | -1.144 |
| | P-value | 0.034 | 0.816 | 0.286 | 0.956 | 0.253 |

| Adeno-associated viruses (AAVs)-based therapies | | | | | | | |
|---|---|---|---|---|---|---|---|
| | | Most likely 1 | 2 | 3 | 4 | Least likely 5 | Total |
| Good knowledge | Number of observations | 8 | 5 | 14 | 7 | 3 | 37 |
| | Frequency | 21.6% | 13.5% | 37.8% | 18.9% | 8.1% | 100.0% |
| High Knowledge | Number of observations | 3 | 5 | 2 | 6 | 9 | 25 |
| | Frequency | 12.0% | 20.0% | 8.0% | 24.0% | 36.0% | 100.0% |

| Lentiviruses-based therapies | | | | | | | |
|---|---|---|---|---|---|---|---|
| | | Most likely 1 | 2 | 3 | 4 | Least likely 5 | Total |
| Good knowledge | Number of observations | 3 | 8 | 5 | 13 | 8 | 37 |
| | Frequency | 8.1% | 21.6% | 13.5% | 35.1% | 21.6% | 100.0% |
| High Knowledge | Number of observations | 3 | 3 | 5 | 10 | 6 | 27 |

| | | | | | | | |
|---|---|---|---|---|---|---|---|
| | **Frequency** | 11.1% | 11.1% | 18.5% | 37.0% | 22.2% | 100.0% |

| **Liposome-based therapies** | | | | | | | |
|---|---|---|---|---|---|---|---|
| | | **Most likely 1** | **2** | **3** | **4** | **Least likely 5** | **Total** |
| **Good knowledge** | **Number of observations** | 5 | 9 | 7 | 5 | 12 | 38 |
| | **Frequency** | 13.2% | 23.7% | 18.4% | 13.2% | 31.6% | 100.0% |
| **High Knowledge** | **Number of observations** | 8 | 5 | 6 | 1 | 8 | 28 |
| | **Frequency** | 28.6% | 17.9% | 21.4% | 3.6% | 28.6% | 100.0% |

| **Messenger RNA (mRNA)-based therapies** | | | | | | | |
|---|---|---|---|---|---|---|---|
| | | **Most likely 1** | **2** | **3** | **4** | **Least likely 5** | **Total** |
| **Good knowledge** | **Number of observations** | 18 | 8 | 7 | 4 | 2 | 39 |
| | **Frequency** | 46.2% | 20.5% | 17.9% | 10.3% | 5.1% | 100.0% |
| **High Knowledge** | **Number of observations** | 11 | 8 | 5 | 2 | 1 | 27 |
| | **Frequency** | 40.7% | 29.6% | 18.5% | 7.4% | 3.7% | 100.0% |

| **Transfer RNA (tRNA)-based therapies** | | | | | | | |
|---|---|---|---|---|---|---|---|
| | | **Most likely 1** | **2** | **3** | **4** | **Least likely 5** | **Total** |
| **Good knowledge** | **Number of observations** | 5 | 9 | 5 | 8 | 12 | 39 |
| | **Frequency** | 12.8% | 23.1% | 12.8% | 20.5% | 30.8% | 100.0% |
| **High Knowledge** | **Number of observations** | 3 | 6 | 8 | 7 | 2 | 26 |
| | **Frequency** | 11.5% | 23.1% | 30.8% | 26.9% | 7.7% | 100.0% |

### 1.4.2 Fixing or replacing the CFTR gene will lead to a cure for cystic fibrosis

| | | | |
|---|---|---|---|
| **Sample size** | | 67 | |
| **Margin of error** | | 11.9% | |
| **Mean** | | 3.46 | |
| **Standard deviation** | | 0.893 | |
| **Percentiles** | ¼ | 3 (likely after 15 years) | |
| | ½ | 4 (likely before 15 years) | |
| | ¾ | 4 (likely before 15 years) | |
| **Mann-Whitney U** | | **Statistic** | **P-value** |
| | | -1.748 | 0.080 |
| **Wilcoxon (H0: median = 4)** | | **Statistic** | **P-value** |
| | | 1.310 | 0.190 |
| **Kolmogorov-Smirnov** | | **Statistic** | **P-value** |
| | | 0.383 | 0.000 |
| **Shapiro-Wilk** | | **Statistic** | **P-value** |
| | | 0.639 | 0.000 |

| | | Unknown | Unlikely | Likely after 15 years | Likely before 15 years | Total |
|---|---|---|---|---|---|---|
| Good knowledge | Number of observations | 5 | 3 | 8 | 23 | 39 |
| | Frequency | 12.8% | 7.7% | 20.5% | 59.0% | 100.0% |
| High Knowledge | Number of observations | 0 | 0 | 7 | 21 | 28 |
| | Frequency | 0.0% | 0.0% | 25.0% | 75.0% | 100.0% |

### 1.4.3 Please rank the following approaches from most likely to least likely to be successful in fixing or replacing the malfunctioning CFTR gene in the next 15 years

| | Most likely (1) | 2 | 3 | Least likely (4) |
|---|---|---|---|---|
| CRISPR-Cas9 | 78.70% | 4.90% | 6.60% | 9.80% |
| TALENs | 12.70% | 52.70% | 25.50% | 9.10% |
| Meganucleases | 5.40% | 21.40% | 28.60% | 44.60% |
| Zinc Finger Nucleases (ZFNs) | 5.40 % | 21.40 % | 37.50% | 35.70% |

| | | CRISPR-Cas9 | TALENs | Meganucleases | Zinc Finger Nucleases (ZFNs) |
|---|---|---|---|---|---|
| Mann-Whitney U | Statistic | -0.516 | -0.305 | -0.289 | -1.143 |
| | P-value | 0.606 | 0.760 | 0.772 | 0.253 |

| CRISPR-Cas9 | | | | | | |
|---|---|---|---|---|---|---|
| | | 1 | 2 | 3 | 4 | Total |
| Good knowledge | Number of observations | 25 | 2 | 3 | 3 | 33 |
| | Frequency | 75.8% | 6.1% | 9.1% | 9.1% | 100.0% |
| High Knowledge | Number of observations | 23 | 1 | 1 | 3 | 28 |
| | Frequency | 82.1% | 3.6% | 3.6% | 10.7% | 100.0% |

| TALENs | | | | | | |
|---|---|---|---|---|---|---|
| | | 1 | 2 | 3 | 4 | Total |
| Good knowledge | Number of observations | 5 | 13 | 9 | 3 | 30 |
| | Frequency | 16.7% | 43.3% | 30.0% | 10.0% | 100.0% |
| High Knowledge | Number of observations | 2 | 16 | 5 | 2 | 25 |
| | Frequency | 8.0% | 64.0% | 20.0% | 8.0% | 100.0% |

| Meganucleases | | | | | | |
|---|---|---|---|---|---|---|
| | | 1 | 2 | 3 | 4 | Total |

| | | | | | | |
|---|---|---|---|---|---|---|
| Good knowledge | Number of observations | 2 | 7 | 6 | 15 | 30 |
| | Frequency | 6.7% | 23.3% | 20.0% | 50.0% | 100.0% |
| High Knowledge | Number of observations | 1 | 5 | 10 | 10 | 26 |
| | Frequency | 3.8% | 19.2% | 38.5% | 38.5% | 100.0% |

| Zinc Finger Nucleases (ZFNs) | | | | | | |
|---|---|---|---|---|---|---|
| | | **1** | **2** | **3** | **4** | **Total** |
| Good knowledge | Number of observations | 1 | 9 | 12 | 9 | 31 |
| | Frequency | 3.2% | 29.0% | 38.7% | 29.0% | 100.0% |
| High Knowledge | Number of observations | 2 | 3 | 9 | 11 | 25 |
| | Frequency | 8.0% | 12.0% | 36.0% | 44.0% | 100.0% |

## 1.5 Respondents who selected 'other' option as the most likely to be successful in treating CF in the next 15 years

### 1.5.1 Fixing or replacing the CFTR gene will lead to a cure for cystic fibrosis

| | | | |
|---|---|---|---|
| **Sample size** | | 24 | |
| **Margin of error** | | 20.0% | |
| **Mean** | | 2.67 | |
| **Standard deviation** | | 1.129 | |
| **Percentiles** | ¼ | 2 (Unlikely) | |
| | ½ | 3 (likely after 15 years) | |
| | ¾ | 4 (likely before 15 years) | |
| **Mann-Whitney U** | | **Statistic** | **P-value** |
| | | -2.190 | 0.028 |
| **Wilcoxon (H0: median = 3)** | | **Statistic** | **P-value** |
| | | -0.502 | 0.616 |
| **Kolmogorov-Smirnov** | | **Statistic** | **P-value** |
| | | 0.199 | 0.015 |
| **Shapiro-Wilk** | | **Statistic** | **P-value** |
| | | 0.857 | 0.003 |

| | | **Unknown** | **Unlikely** | **Likely after 15 years** | **Likely before 15 years** | **Total** |
|---|---|---|---|---|---|---|
| Good knowledge | Number of observations | 5 | 4 | 3 | 3 | 15 |
| | Frequency | 33.3% | 26.7% | 20.0% | 20.0% | 100.0% |
| High Knowledge | Number of observations | 0 | 1 | 4 | 4 | 9 |
| | Frequency | 0.0% | 11.1% | 44.4% | 44.4% | 100.0% |

### 1.5.2 Please rank the following approaches from most likely to least likely to be successful in fixing or replacing the malfunctioning CFTR gene in the next 15 years

| | Most likely (1) | 2 | 3 | Least likely (4) |
|---|---|---|---|---|
| **CRISPR-Cas9** | 50,00% | 0,00% | 12,50% | 37,50% |
| **TALENs** | 16,70% | 33,30% | 50,00% | 0,00% |
| **Meganucleases** | 0,00% | 16,70% | 33,30% | 50,00% |
| **Zinc Finger Nucleases (ZFNs)** | 33,30% | 50,00% | 0,00% | 16,70% |

| | | CRISPR-Cas9 | TALENs | Meganucleases | Zinc Finger Nucleases (ZFNs) |
|---|---|---|---|---|---|
| **Mann-Whitney U** | **Statistic** | -2.449 | -1.000 | -1.000 | -2.000 |
| | **P-value** | 0.014 | 0.317 | 0.317 | 0.046 |

| CRISPR-Cas9 | | | | | | |
|---|---|---|---|---|---|---|
| | | **1** | **2** | **3** | **4** | **Total** |
| **Good knowledge** | **Number of observations** | 0 | 0 | 0 | 3 | 3 |
| | **Frequency** | 0.0% | 0.0% | 0.0% | 100.0% | 100.0% |
| **High Knowledge** | **Number of observations** | 4 | 0 | 1 | 0 | 5 |
| | **Frequency** | 80.0% | 0.0% | 20.0% | 0.0% | 100.0% |

| TALENs | | | | | | |
|---|---|---|---|---|---|---|
| | | **1** | **2** | **3** | **4** | **Total** |
| **Good knowledge** | **Number of observations** | 0 | 2 | 0 | 0 | 2 |
| | **Frequency** | 0.0% | 100.0% | 0.0% | 0.0% | 100.0% |
| **High Knowledge** | **Number of observations** | 1 | 0 | 3 | 0 | 4 |
| | **Frequency** | 25.0% | 0.0% | 75.0% | 0.0% | 100.0% |

| Meganucleases | | | | | | |
|---|---|---|---|---|---|---|
| | | **1** | **2** | **3** | **4** | **Total** |
| **Good knowledge** | **Number of observations** | 0 | 0 | 2 | 0 | 2 |
| | **Frequency** | 0.0% | 0.0% | 100.0% | 0.0% | 100.0% |
| **High Knowledge** | **Number of observations** | 0 | 1 | 0 | 3 | 4 |
| | **Frequency** | 0.0% | 25.0% | 0.0% | 75.0% | 100.0% |

| Zinc Finger Nucleases (ZFNs) | | | | | | |
|---|---|---|---|---|---|---|
| | | **1** | **2** | **3** | **4** | **Total** |
| **Good knowledge** | **Number of observations** | 2 | 0 | 0 | 0 | 2 |

| | | | | | | |
|---|---|---|---|---|---|---|
| | **Frequency** | 100.0% | 0.0% | 0.0% | 0.0% | 100.0% |
| **High Knowledge** | **Number of observations** | 0 | 3 | 0 | 1 | 4 |
| | **Frequency** | 0.0% | 75.0% | 0.0% | 25.0% | 100.0% |

# 2 Descriptive statistics of the results according to the chosen therapeutic option and the respondents' level of knowledge

## 2.1 Respondents who selected CFTR modulator therapies as the most likely to be successful in treating CF in the next 15 years

### 2.1.1 Please rank the following approaches from most likely to least likely to be successful in treating cystic fibrosis in the next 15 years

**Potentiators**

| | 1 | | 2 | | 3 | | 4 | | Total | | Score |
|---|---|---|---|---|---|---|---|---|---|---|---|
| Q1: I have high knowledge | 26.77% | 34 | 58.27% | 74 | 8.66% | 11 | 6.30% | 8 | 43.49% | 127 | 3.06 |
| Q1: I have good knowledge | 24.17% | 29 | 44.17% | 53 | 20.83% | 25 | 10.83% | 13 | 41.10% | 120 | 2.82 |
| Q1: I have some knowledge | 17.95% | 7 | 43.59% | 17 | 25.64% | 10 | 12.82% | 5 | 13.36% | 39 | 2.67 |
| Total | 23.97% | 70 | 49.32% | 144 | 15.75% | 46 | 8.90% | 26 | 100.00% | 292 | |
| | | | | | | | | | **Answered** | **292** | |

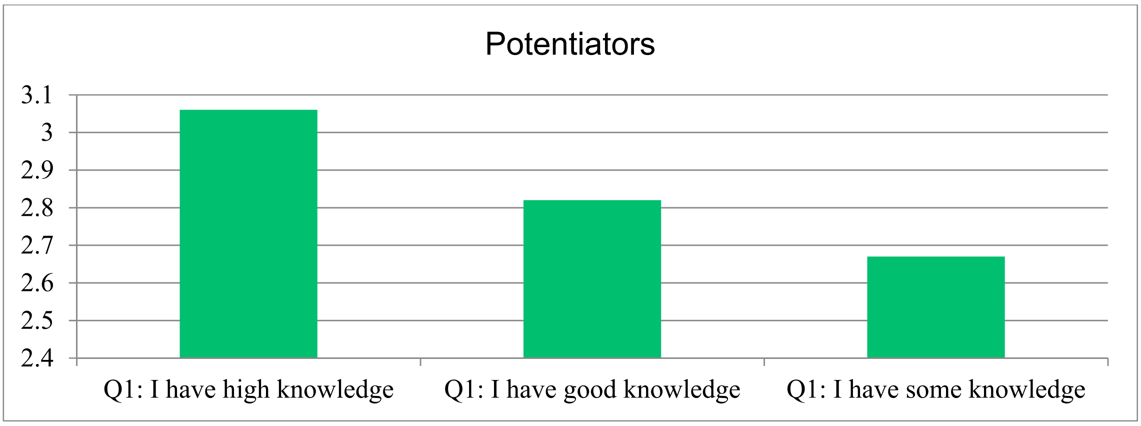


**Correctors**

| | 1 | | 2 | | 3 | | 4 | | Total | | Score |
|---|---|---|---|---|---|---|---|---|---|---|---|
| Q1: I have high knowledge | 62.50% | 80 | 23.44% | 30 | 6.25% | 8 | 7.81% | 10 | 43.84% | 128 | 3.41 |
| Q1: I have good knowledge | 51.24% | 62 | 23.14% | 28 | 12.40% | 15 | 13.22% | 16 | 41.44% | 121 | 3.12 |
| Q1: I have some knowledge | 39.47% | 15 | 18.42% | 7 | 21.05% | 8 | 21.05% | 8 | 13.01% | 38 | 2.76 |
| Total | 53.77% | 157 | 22.26% | 65 | 10.62% | 31 | 11.64% | 34 | 100.00% | 292 | |
| | | | | | | | | | **Answered** | **292** | |

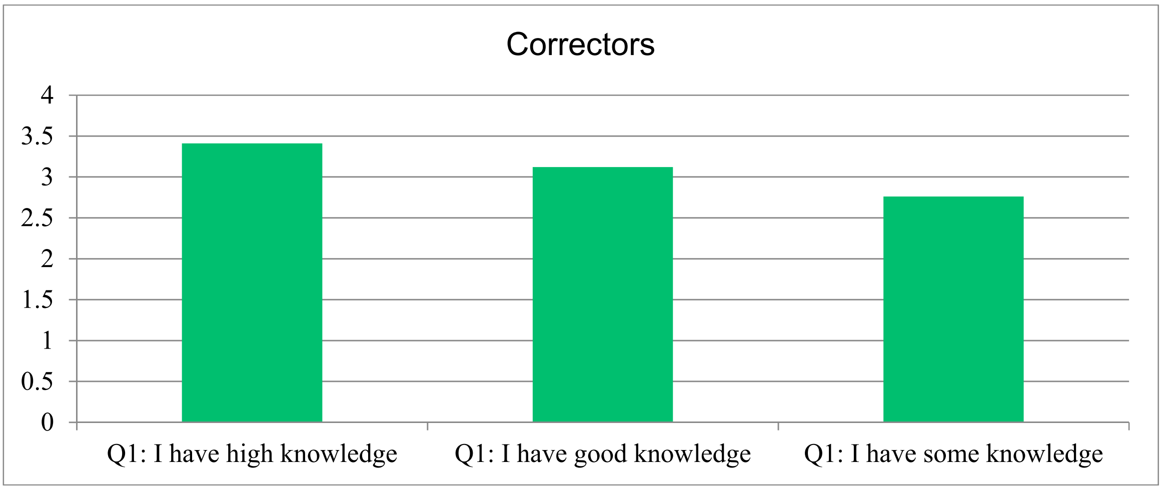


## Amplifiers

| | 1 | | 2 | | 3 | | 4 | | Total | | Score |
|---|---|---|---|---|---|---|---|---|---|---|---|
| Q1: I have high knowledge | 8.06% | 10 | 9.68% | 12 | 49.19% | 61 | 33.06% | 41 | 42.47% | 124 | 1.93 |
| Q1: I have good knowledge | 8.62% | 10 | 15.52% | 18 | 36.21% | 42 | 39.66% | 46 | 39.73% | 116 | 1.93 |
| Q1: I have some knowledge | 13.89% | 5 | 22.22% | 8 | 22.22% | 8 | 41.67% | 15 | 12.33% | 36 | 2.08 |
| Total | 8.56% | 25 | 13.01% | 38 | 38.01% | 111 | 34.93% | 102 | 100.00% | 292 | |
| | | | | | | | | | | **Answered** | **292** |

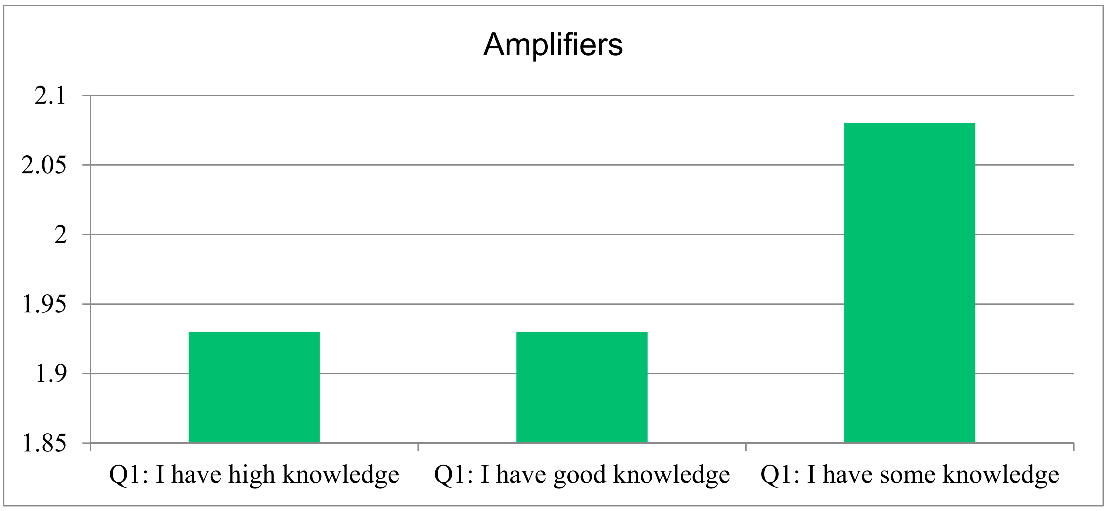


## Stabilizers

| | 1 | | 2 | | 3 | | 4 | | Total | | Score |
|---|---|---|---|---|---|---|---|---|---|---|---|
| Q1: I have high knowledge | 2.40% | 3 | 8.80% | 11 | 36.00% | 45 | 52.80% | 66 | 42.81% | 125 | 1.61 |
| Q1: I have good knowledge | 16.38% | 19 | 16.38% | 19 | 31.90% | 37 | 35.34% | 41 | 39.73% | 116 | 2.14 |
| Q1: I have some knowledge | 26.32% | 10 | 15.79% | 6 | 31.58% | 12 | 26.32% | 10 | 13.01% | 38 | 2.42 |
| Total | 10.96% | 32 | 12.33% | 36 | 32.19% | 94 | 40.07% | 117 | 100.00% | 292 | |
| | | | | | | | | | | **Answered** | **292** |

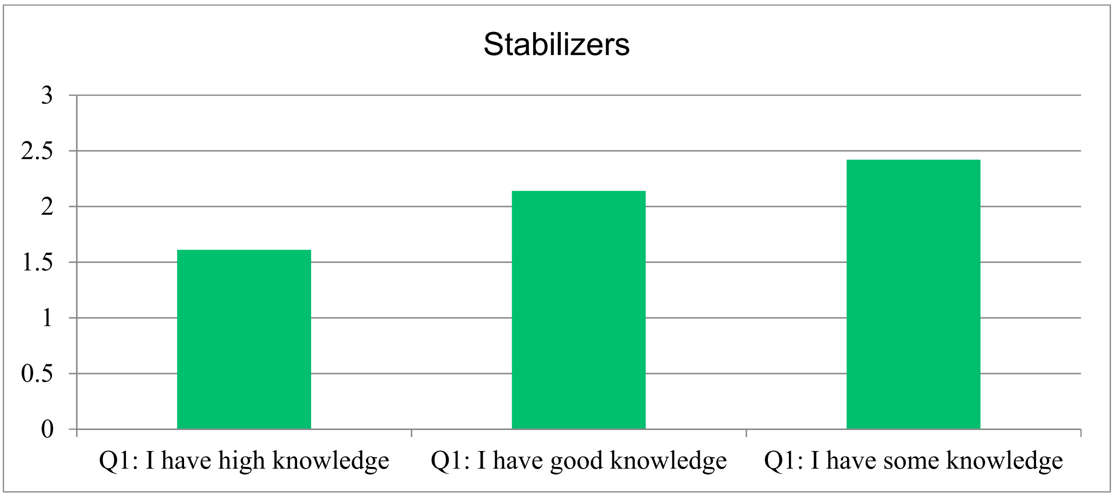


### 2.1.2 Fixing or replacing the CFTR gene will lead to a cure for cystic fibrosis

| | Likely before 15 years | | Likely after 15 years | | Unlikely | | Unknown | | Total | |
|---|---|---|---|---|---|---|---|---|---|---|
| Q1: I have high knowledge | 26.36% | 34 | 50.39% | 65 | 10.85% | 14 | 12.40% | 16 | 43.43% | 129 |
| Q1: I have good knowledge | 28.46% | 35 | 43.90% | 54 | 7.32% | 9 | 20.33% | 25 | 41.41% | 123 |
| Q1: I have some knowledge | 28.89% | 13 | 42.22% | 19 | 6.67% | 3 | 22.22% | 10 | 15.15% | 45 |
| Total | 27.61% | 82 | 46.46% | 138 | 8.75% | 26 | 17.17% | 51 | 100.00% | 297 |
| | | | | | | | | | **Answered** | **297** |

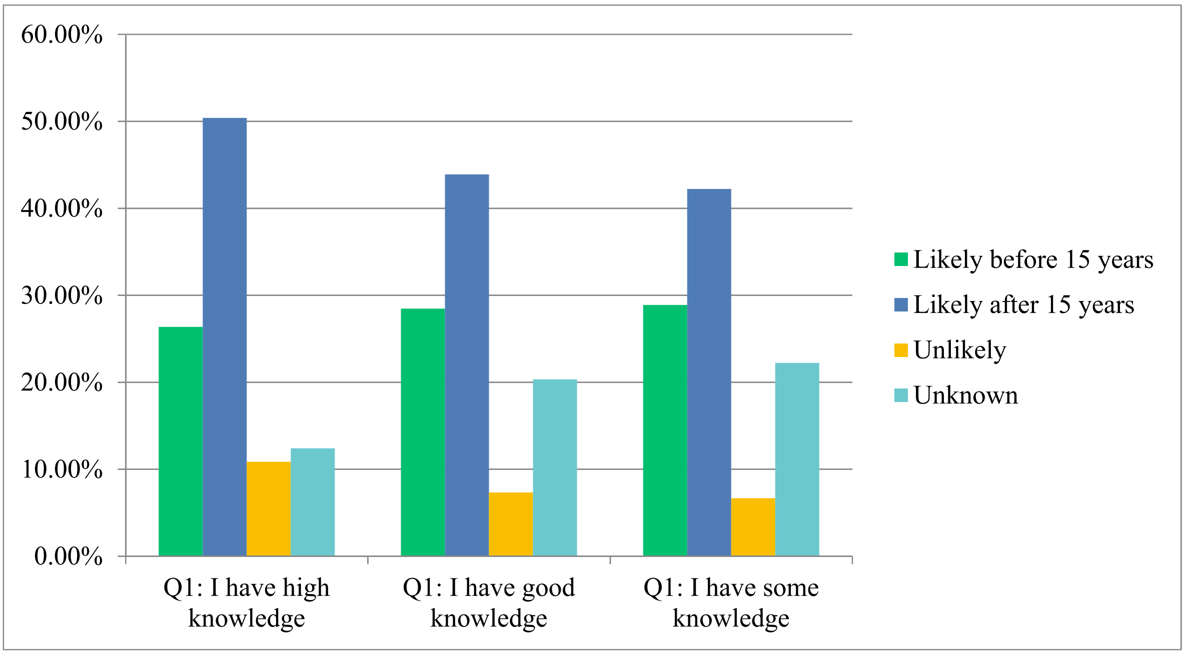


### 2.1.3 Please rank the following approaches from most likely to least likely to be successful in fixing or replacing the malfunctioning CFTR gene in the next 15 years

**CRISPR-Cas9**

| | 1 | | 2 | | 3 | | 4 | | Total | | Score |
|---|---|---|---|---|---|---|---|---|---|---|---|
| Q1: I have high knowledge | 78.31% | 65 | 10.84% | 9 | 4.82% | 4 | 6.02% | 5 | 45.86% | 83 | 3.61 |
| Q1: I have good knowledge | 66.20% | 47 | 15.49% | 11 | 2.82% | 2 | 15.49% | 11 | 39.23% | 71 | 3.32 |
| Q1: I have some knowledge | 70.83% | 17 | 8.33% | 2 | 8.33% | 2 | 12.50% | 3 | 13.26% | 24 | 3.38 |
| Total | 71.27% | 129 | 12.15% | 22 | 4.42% | 8 | 10.50% | 19 | 100.00% | 181 | |
| | | | | | | | | | | **Answered** | **181** |

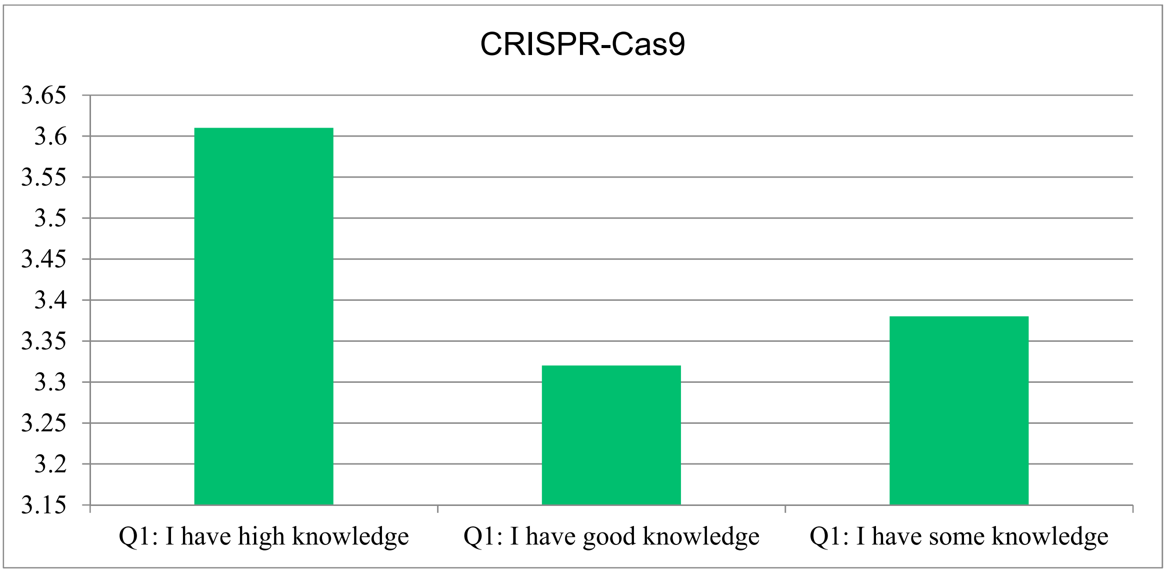


## TALENs

| | 1 | | 2 | | 3 | | 4 | | Total | | Score |
|---|---|---|---|---|---|---|---|---|---|---|---|
| Q1: I have high knowledge | 12.20% | 10 | 41.46% | 34 | 29.27% | 24 | 17.07% | 14 | 45.30% | 82 | 2.49 |
| Q1: I have good knowledge | 17.91% | 12 | 46.27% | 31 | 23.88% | 16 | 11.94% | 8 | 37.02% | 67 | 2.7 |
| Q1: I have some knowledge | 19.05% | 4 | 33.33% | 7 | 23.81% | 5 | 23.81% | 5 | 11.60% | 21 | 2.48 |
| Total | 14.36% | 26 | 39.78% | 72 | 24.86% | 45 | 14.92% | 27 | 100.00% | 181 | |
| | | | | | | | | | | **Answered** | **181** |

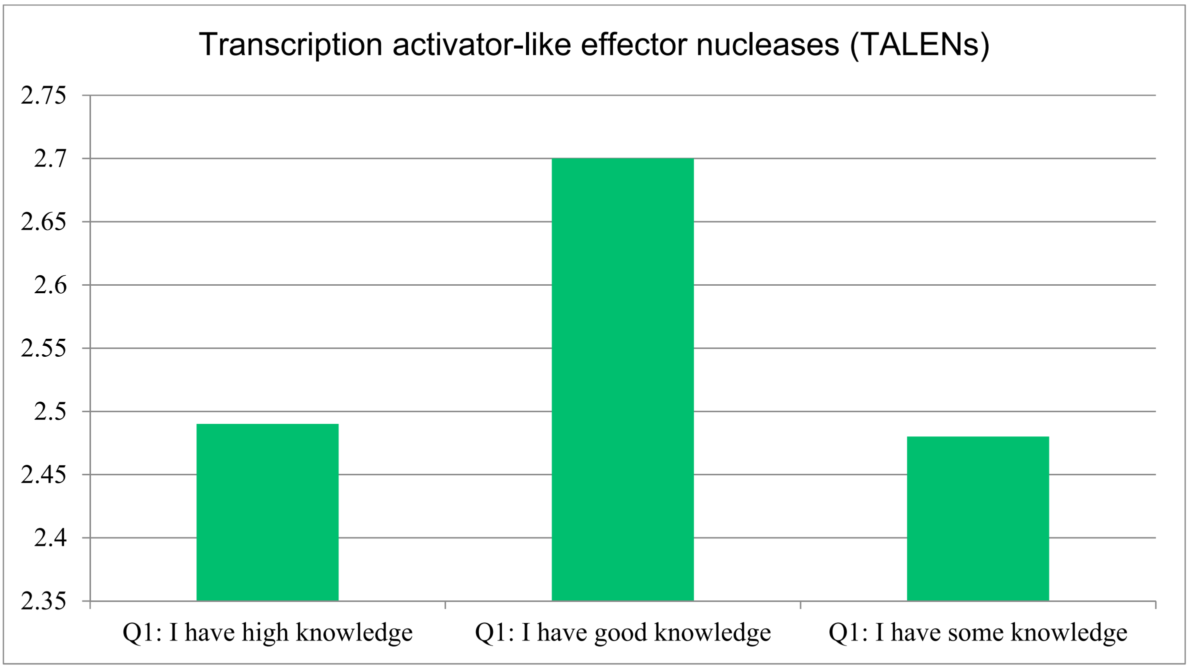


## Meganucleases (MNs)

| | 1 | | 2 | | 3 | | 4 | | Total | | Score |
|---|---|---|---|---|---|---|---|---|---|---|---|
| Q1: I have high knowledge | 6.25% | 5 | 13.75% | 11 | 35.00% | 28 | 45.00% | 36 | 44.20% | 80 | 1.81 |
| Q1: I have good knowledge | 10.29% | 7 | 8.82% | 6 | 36.76% | 25 | 44.12% | 30 | 37.57% | 68 | 1.85 |
| Q1: I have some knowledge | 0.00% | 0 | 31.82% | 7 | 40.91% | 9 | 27.27% | 6 | 12.15% | 22 | 2.05 |
| Total | 6.63% | 12 | 13.26% | 24 | 34.25% | 62 | 39.78% | 72 | 100.00% | 181 | |
| | | | | | | | | | | **Answered** | **181** |

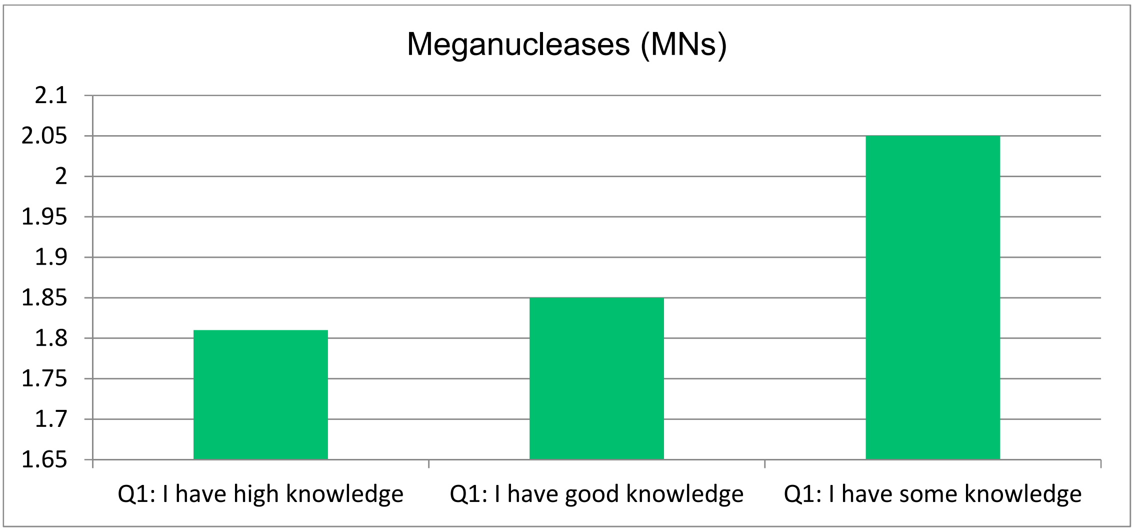

**Zinc finger nucleases (ZFNs)**

| | 1 | | 2 | | 3 | | 4 | | Total | | Score |
|---|---|---|---|---|---|---|---|---|---|---|---|
| Q1: I have high knowledge | 3.70% | 3 | 33.33% | 27 | 32.10% | 26 | 30.86% | 25 | 44.75% | 81 | 2.1 |
| Q1: I have good knowledge | 7.46% | 5 | 28.36% | 19 | 37.31% | 25 | 26.87% | 18 | 37.02% | 67 | 2.16 |
| Q1: I have some knowledge | 9.52% | 2 | 28.57% | 6 | 23.81% | 5 | 38.10% | 8 | 11.60% | 21 | 2.1 |
| Total | 5.52% | 10 | 28.73% | 52 | 30.94% | 56 | 28.18% | 51 | 100.00% | 181 | |
| | | | | | | | | | | **Answered** | **181** |

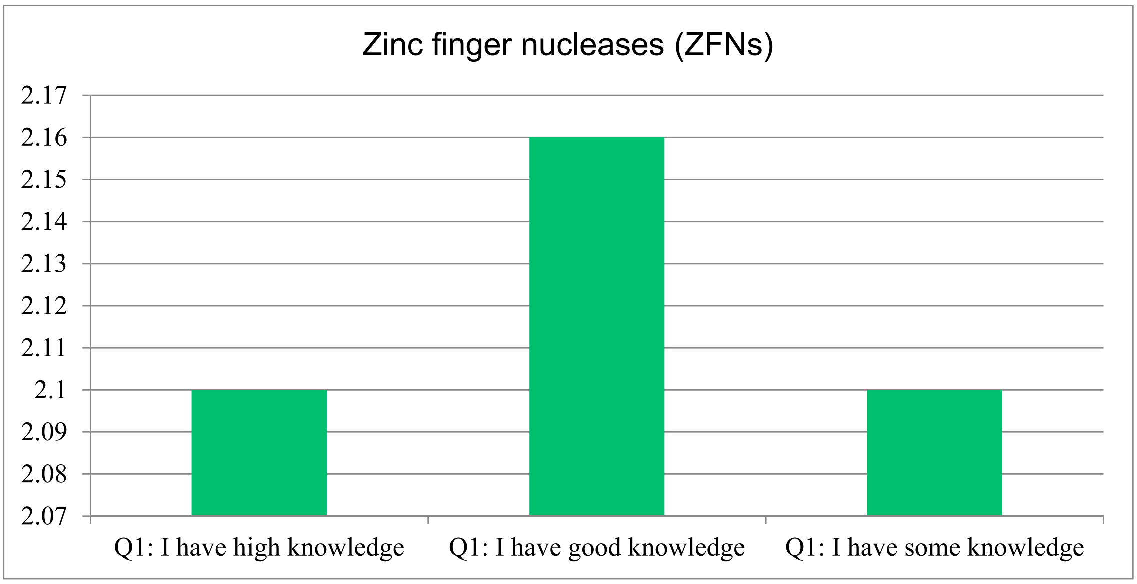


## 2.2 Respondents who selected genetic therapies as the most likely to be successful in treating CF in the next 15 years

### 2.2.1 Please rank the following therapies from most likely to least likely to be successful in treating CF in the next 15 years

**AAVs-based therapies**

| | 1 | | 2 | | 3 | | 4 | | 5 | | Total | | Score |
|---|---|---|---|---|---|---|---|---|---|---|---|---|---|
| Q1: I have high knowledge | 12.00% | 3 | 20.00% | 5 | 8.00% | 2 | 24.00% | 6 | 36.00% | 9 | 27.17% | 25 | 2.48 |
| Q1: I have good knowledge | 22.22% | 8 | 13.89% | 5 | 36.11% | 13 | 19.44% | 7 | 8.33% | 3 | 39.13% | 36 | 3.22 |
| Q1: I have some knowledge | 26.09% | 6 | 26.09% | 6 | 8.70% | 2 | 21.74% | 5 | 17.39% | 4 | 25.00% | 23 | 3.22 |
| Total | 18.48% | 17 | 17.39% | 16 | 18.48% | 17 | 19.57% | 18 | 17.39% | 16 | 100.00% | 92 | |
| | | | | | | | | | | | | **Answered** | **92** |

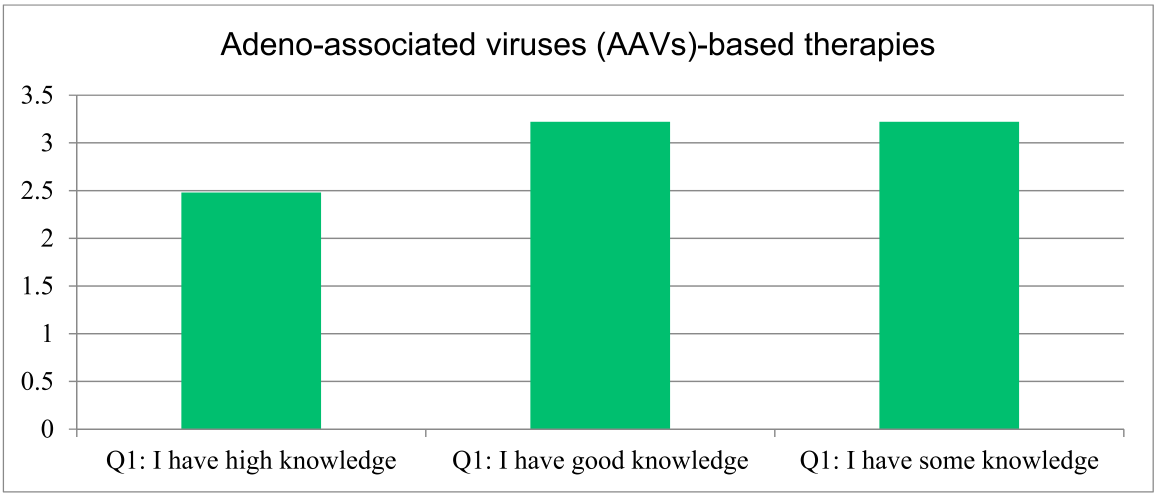


### Lentiviruses-bases therapies

| | 1 | | 2 | | 3 | | 4 | | 5 | | Total | | Score |
|---|---|---|---|---|---|---|---|---|---|---|---|---|---|
| Q1: I have high knowledge | 11.11% | 3 | 11.11% | 3 | 18.52% | 5 | 37.04% | 10 | 22.22% | 6 | 29.35% | 27 | 2.52 |
| Q1: I have good knowledge | 8.33% | 3 | 22.22% | 8 | 13.89% | 5 | 36.11% | 13 | 19.44% | 7 | 39.13% | 36 | 2.64 |
| Q1: I have some knowledge | 22.73% | 5 | 9.09% | 2 | 18.18% | 4 | 36.36% | 8 | 13.64% | 3 | 23.91% | 22 | 2.91 |
| Total | 11.96% | 11 | 14.13% | 13 | 15.22% | 14 | 33.70% | 31 | 17.39% | 16 | 100.00% | 92 | |
| | | | | | | | | | | | | **Answered** | **92** |

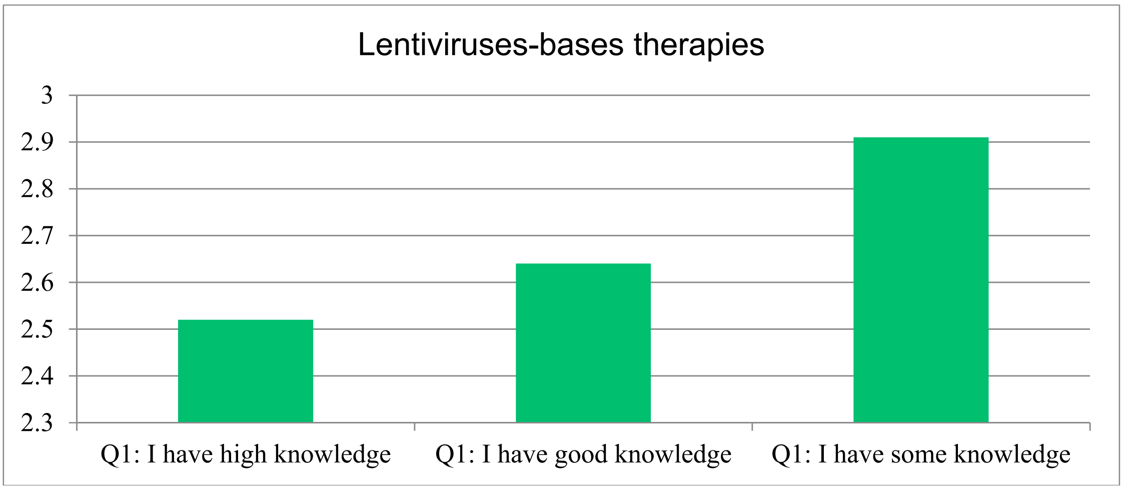


### Liposome-based therapies

| | 1 | | 2 | | 3 | | 4 | | 5 | | Total | | Score |
|---|---|---|---|---|---|---|---|---|---|---|---|---|---|
| Q1: I have high knowledge | 28.57% | 8 | 17.86% | 5 | 21.43% | 6 | 3.57% | 1 | 28.57% | 8 | 30.43% | 28 | 3.14 |
| Q1: I have good knowledge | 13.51% | 5 | 21.62% | 8 | 18.92% | 7 | 13.51% | 5 | 32.43% | 12 | 40.22% | 37 | 2.7 |
| Q1: I have some knowledge | 13.04% | 3 | 21.74% | 5 | 30.43% | 7 | 17.39% | 4 | 17.39% | 4 | 25.00% | 23 | 2.96 |
| Total | 17.39% | 16 | 19.57% | 18 | 21.74% | 20 | 10.87% | 10 | 26.09% | 24 | 100.00% | 92 | |
| | | | | | | | | | | | | **Answered** | **92** |

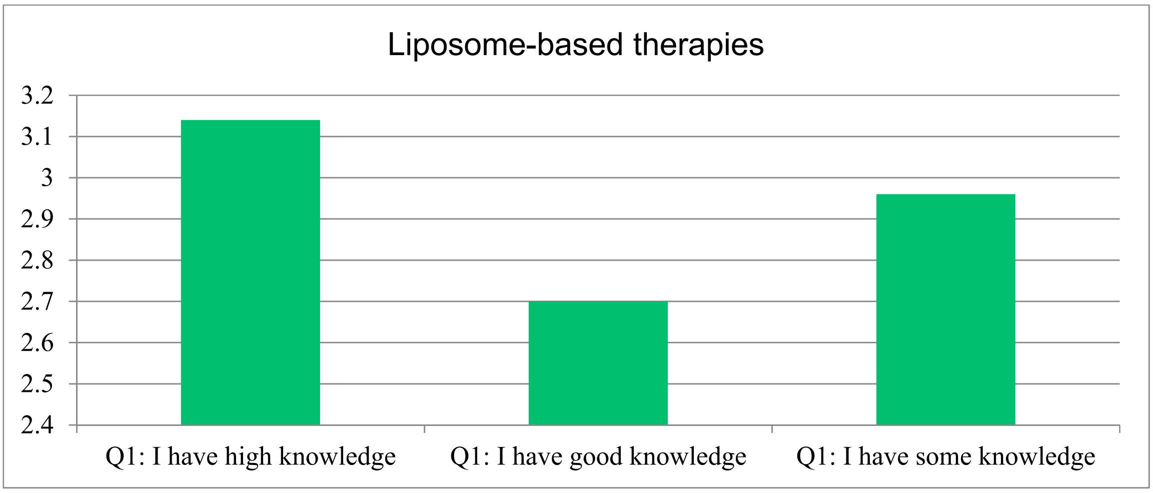


## mRNA-based therapies

| | 1 | | 2 | | 3 | | 4 | | 5 | | Total | | Score |
|---|---|---|---|---|---|---|---|---|---|---|---|---|---|
| Q1: I have high knowledge | 40.74% | 11 | 29.63% | 8 | 18.52% | 5 | 7.41% | 2 | 3.70% | 1 | 29.35% | 27 | 3.96 |
| Q1: I have good knowledge | 44.74% | 17 | 21.05% | 8 | 18.42% | 7 | 10.53% | 4 | 5.26% | 2 | 41.30% | 38 | 3.89 |
| Q1: I have some knowledge | 34.62% | 9 | 19.23% | 5 | 11.54% | 3 | 15.38% | 4 | 19.23% | 5 | 28.26% | 26 | 3.35 |
| Total | 40.22% | 37 | 22.83% | 21 | 16.30% | 15 | 10.87% | 10 | 8.70% | 8 | 100.00% | 92 | |

**Answered** **92**

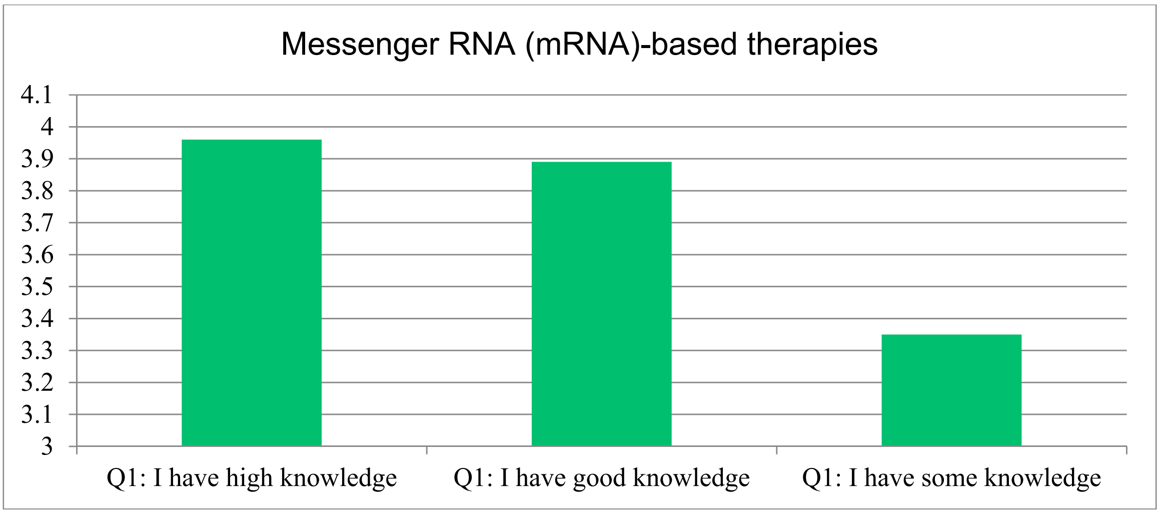


**tRNA-based therapies**

| | 1 | | 2 | | 3 | | 4 | | 5 | | Total | | Score |
|---|---|---|---|---|---|---|---|---|---|---|---|---|---|
| Q1: I have high knowledge | 11.54% | 3 | 23.08% | 6 | 30.77% | 8 | 26.92% | 7 | 7.69% | 2 | 28.26% | 26 | 3.04 |
| Q1: I have good knowledge | 13.16% | 5 | 23.68% | 9 | 13.16% | 5 | 18.42% | 7 | 31.58% | 12 | 41.30% | 38 | 2.68 |
| Q1: I have some knowledge | 4.76% | 1 | 23.81% | 5 | 28.57% | 6 | 4.76% | 1 | 38.10% | 8 | 22.83% | 21 | 2.52 |
| Total | 9.78% | 9 | 21.74% | 20 | 20.65% | 19 | 16.30% | 15 | 23.91% | 22 | 100.00% | 92 | |

**Answered** **92**

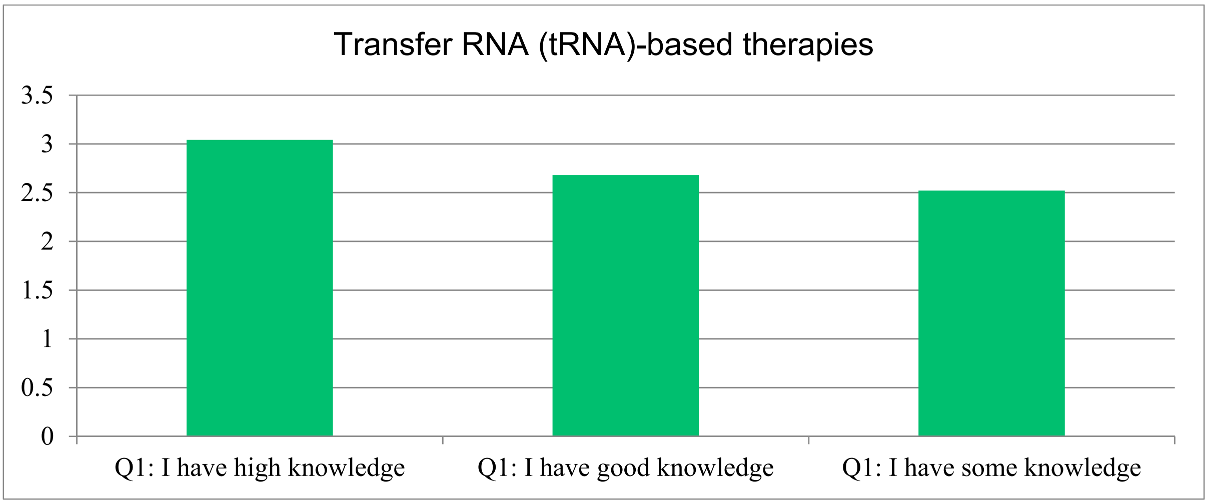


### 2.2.2 Fixing or replacing the CFTR gene will lead to a cure for cystic fibrosis

| | Likely before 15 years | | Likely after 15 years | | Unlikely | | Unknown | | Total | |
|---|---|---|---|---|---|---|---|---|---|---|
| Q1: I have high knowledge | 75.00% | 21 | 25.00% | 7 | 0.00% | 0 | 0.00% | 0 | 30.11% | 28 |
| Q1: I have good knowledge | 58.97% | 23 | 20.51% | 8 | 7.69% | 3 | 12.82% | 5 | 41.94% | 39 |
| Q1: I have some knowledge | 61.54% | 16 | 23.08% | 6 | 0.00% | 0 | 15.38% | 4 | 27.96% | 26 |
| Total | 64.52% | 60 | 22.58% | 21 | 3.23% | 3 | 9.68% | 9 | 100.00% | 93 |
| | | | | | | | | | **Answered** | **93** |

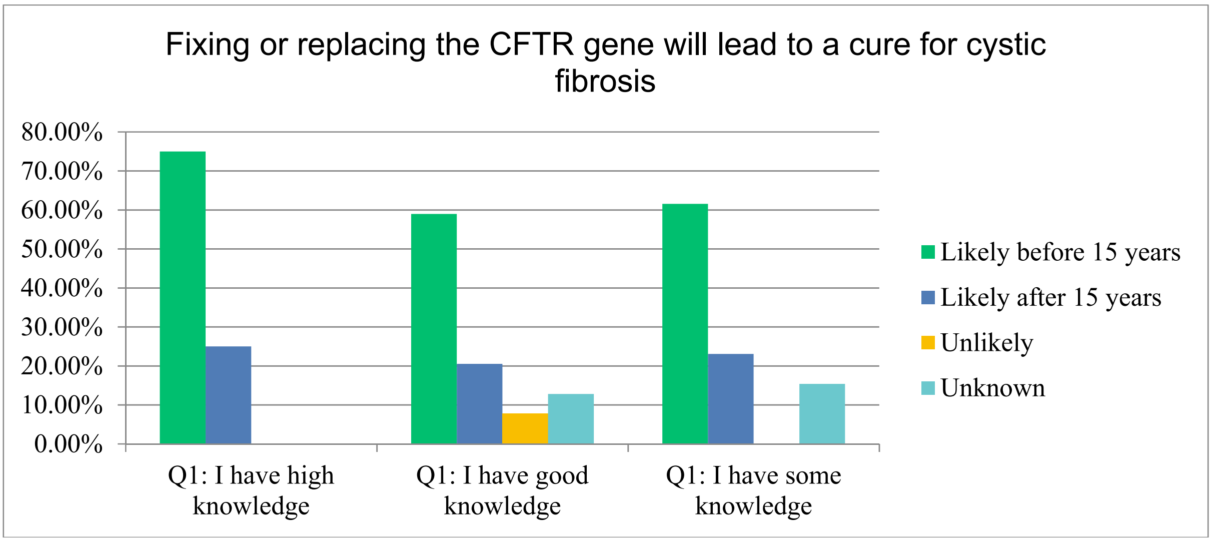


### 2.2.3 Please rank the following approaches from most likely to least likely to be successful in fixing or replacing the malfunctioning CFTR gene in the next 15 years

**CRISPR-Cas9**

| | 1 | | 2 | | 3 | | 4 | | Total | | Score |
|---|---|---|---|---|---|---|---|---|---|---|---|
| Q1: I have high knowledge | 82.14% | 23 | 3.57% | 1 | 3.57% | 1 | 10.71% | 3 | 32.94% | 28 | 3.57 |
| Q1: I have good knowledge | 75.76% | 25 | 6.06% | 2 | 9.09% | 3 | 9.09% | 3 | 38.82% | 33 | 3.48 |
| Q1: I have some knowledge | 69.57% | 16 | 13.04% | 3 | 8.70% | 2 | 8.70% | 2 | 27.06% | 23 | 3.43 |

| | 1 | | 2 | | 3 | | 4 | | Total | | Score |
|---|---|---|---|---|---|---|---|---|---|---|---|
| Total | 75.29% | 64 | 7.06% | 6 | 7.06% | 6 | 9.41% | 8 | 100.00% | 85 | |
| | | | | | | | | | | Answered | 85 |

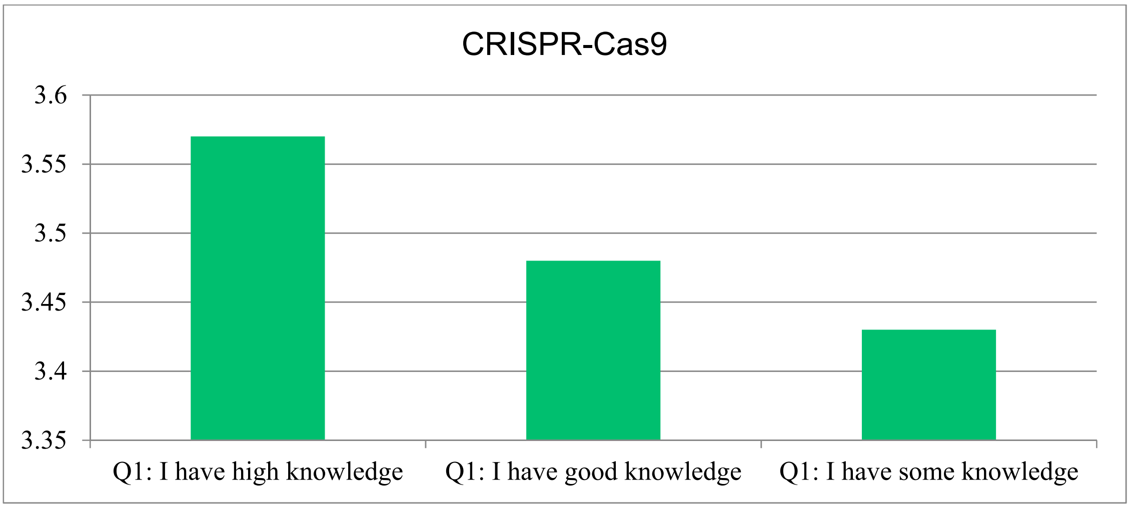


## TALENs

| | 1 | | 2 | | 3 | | 4 | | Total | | Score |
|---|---|---|---|---|---|---|---|---|---|---|---|
| Q1: I have high knowledge | 8.00% | 2 | 64.00% | 16 | 20.00% | 5 | 8.00% | 2 | 29.41% | 25 | 2.72 |
| Q1: I have good knowledge | 16.67% | 5 | 43.33% | 13 | 30.00% | 9 | 10.00% | 3 | 35.29% | 30 | 2.67 |
| Q1: I have some knowledge | 5.56% | 1 | 61.11% | 11 | 16.67% | 3 | 16.67% | 3 | 21.18% | 18 | 2.56 |
| Total | 9.41% | 8 | 47.06% | 40 | 20.00% | 17 | 9.41% | 8 | 100.00% | 85 | |
| | | | | | | | | | | Answered | 85 |

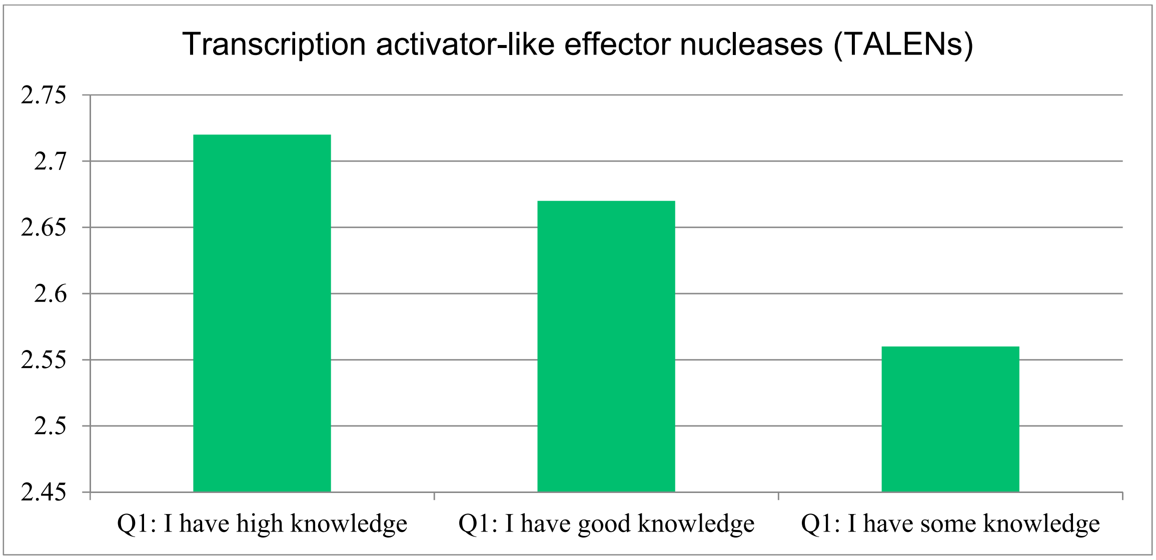


## Meganucleases (MNs)

| | 1 | | 2 | | 3 | | 4 | | Total | | Score |
|---|---|---|---|---|---|---|---|---|---|---|---|
| Q1: I have high knowledge | 3.85% | 1 | 19.23% | 5 | 38.46% | 10 | 38.46% | 10 | 30.59% | 26 | 1.88 |
| Q1: I have good knowledge | 6.67% | 2 | 23.33% | 7 | 20.00% | 6 | 50.00% | 15 | 35.29% | 30 | 1.87 |
| Q1: I have some knowledge | 13.33% | 2 | 13.33% | 2 | 26.67% | 4 | 46.67% | 7 | 17.65% | 15 | 1.93 |
| Total | 5.88% | 5 | 16.47% | 14 | 23.53% | 20 | 37.65% | 32 | 100.00% | 85 | |
| | | | | | | | | | | Answered | 85 |

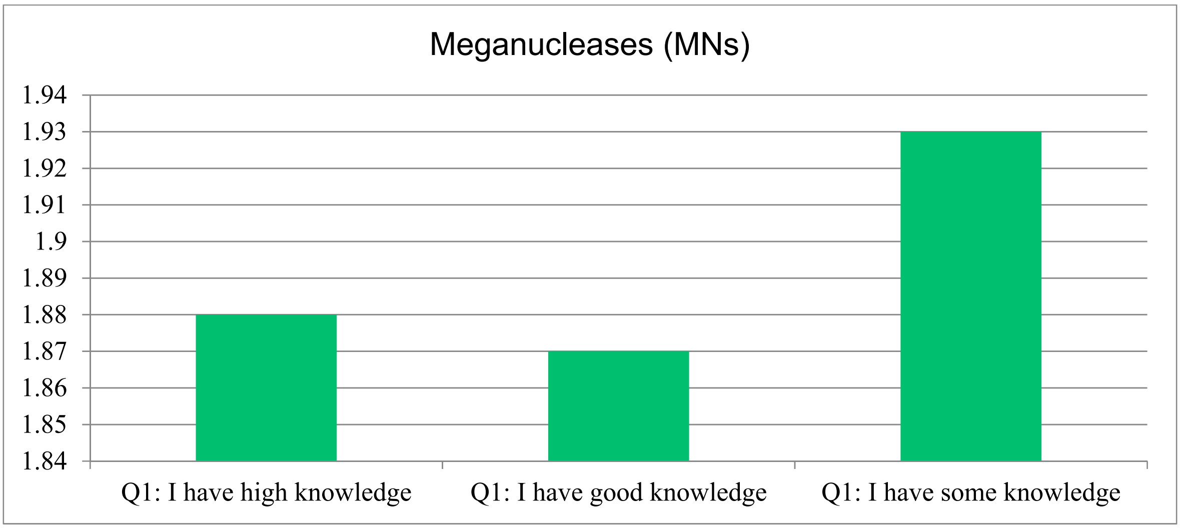


## Zinc finger nucleases (ZFNs)

| | 1 | | 2 | | 3 | | 4 | | Total | | Score |
|---|---|---|---|---|---|---|---|---|---|---|---|
| Q1: I have high knowledge | 8.00% | 2 | 12.00% | 3 | 36.00% | 9 | 44.00% | 11 | 29.41% | 25 | 1.84 |
| Q1: I have good knowledge | 3.23% | 1 | 29.03% | 9 | 38.71% | 12 | 29.03% | 9 | 36.47% | 31 | 2.06 |
| Q1: I have some knowledge | 11.76% | 2 | 17.65% | 3 | 47.06% | 8 | 23.53% | 4 | 20.00% | 17 | 2.18 |
| Total | 5.88% | 5 | 17.65% | 15 | 34.12% | 29 | 28.24% | 24 | 100.00% | 85 | |

**Answered** 85

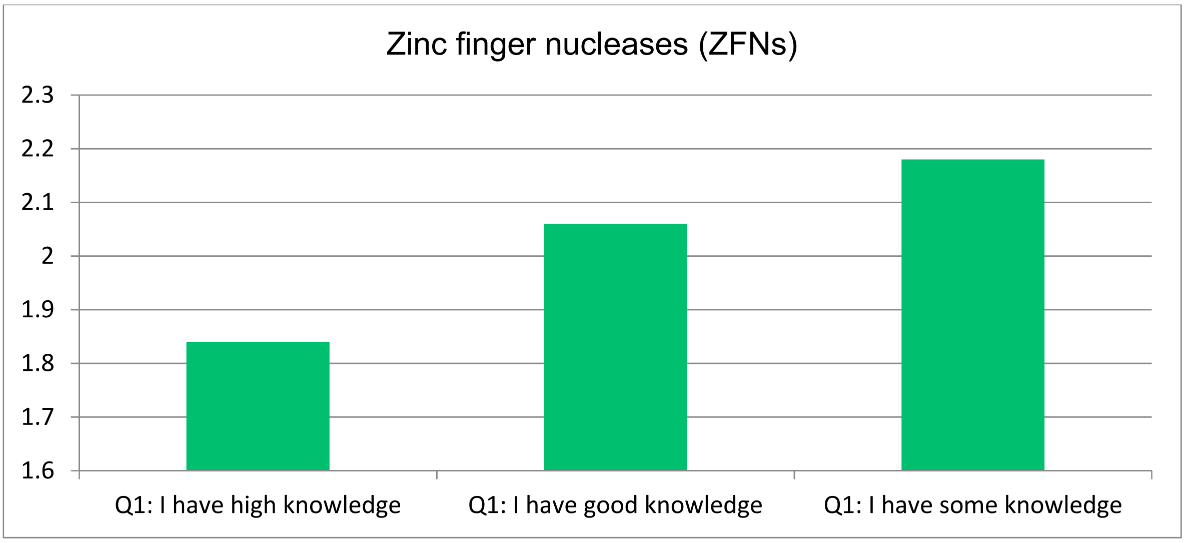

## 2.3 Respondents who selected 'other' option as the most likely to be successful in treating CF in the next 15 years

### 2.3.1 Fixing or replacing the CFTR gene will lead to a cure for cystic fibrosis

| | Likely before 15 years | | Likely after 15 years | | Unlikely | | Unknown | | Total | |
|---|---|---|---|---|---|---|---|---|---|---|
| Q1: I have high knowledge | 44.44% | 4 | 44.44% | 4 | 11.11% | 1 | 0.00% | 0 | 30.00% | 9 |
| Q1: I have good knowledge | 14.29% | 2 | 21.43% | 3 | 28.57% | 4 | 35.71% | 5 | 46.67% | 14 |
| Q1: I have some knowledge | 28.57% | 2 | 0.00% | 0 | 28.57% | 2 | 42.86% | 3 | 23.33% | 7 |
| Total | 26.67% | 8 | 23.33% | 7 | 23.33% | 7 | 26.67% | 8 | 100.00% | 30 |
| | | | | | | | | | **Answered** | **30** |

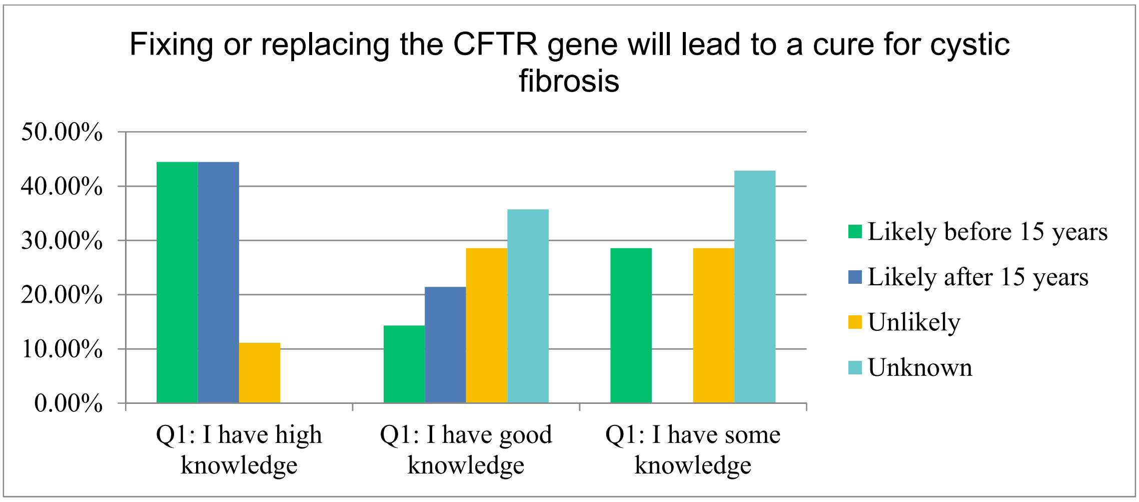


### 2.3.2 Please rank the following approaches from most likely to least likely to be successful in fixing or replacing the malfunctioning CFTR gene in the next 15 years

**CRISPR-Cas9**

| | 1 | | 2 | | 3 | | 4 | | Total | | Score |
|---|---|---|---|---|---|---|---|---|---|---|---|
| Q1: I have high knowledge | 80.00% | 4 | 0.00% | 0 | 20.00% | 1 | 0.00% | 0 | 55.56% | 5 | 3.6 |
| Q1: I have good knowledge | 0.00% | 0 | 0.00% | 0 | 0.00% | 0 | 100.00% | 3 | 33.33% | 3 | 1 |
| Q1: I have some knowledge | 100.00% | 1 | 0.00% | 0 | 0.00% | 0 | 0.00% | 0 | 11.11% | 1 | 4 |
| Total | 55.56% | 5 | 0.00% | 0 | 11.11% | 1 | 33.33% | 3 | 100.00% | 9 | |
| | | | | | | | | | | **Answered** | **9** |

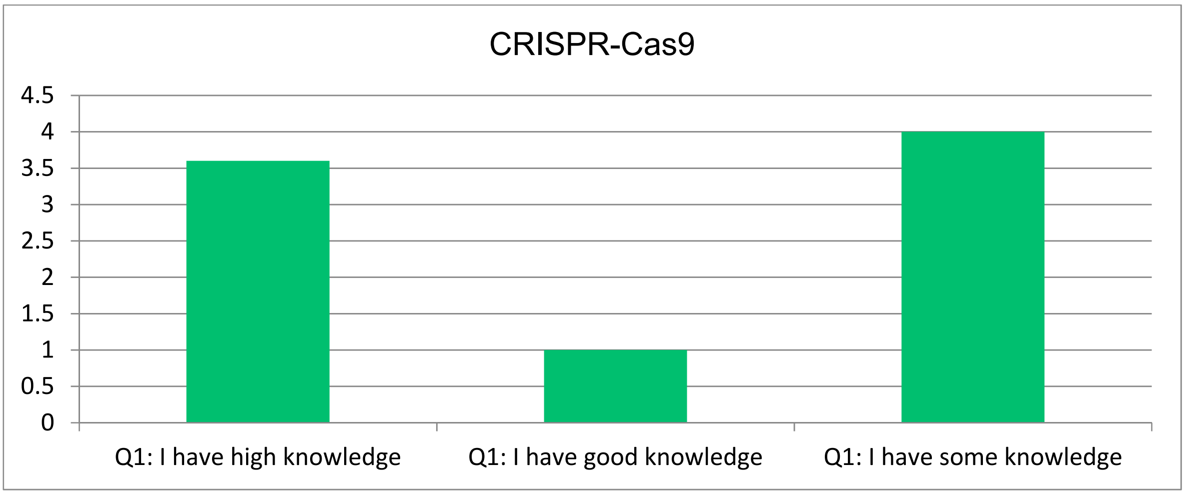


## TALENs

| | 1 | | 2 | | 3 | | 4 | | Total | | Score |
|---|---|---|---|---|---|---|---|---|---|---|---|
| Q1: I have high knowledge | 25.00% | 1 | 0.00% | 0 | 75.00% | 3 | 0.00% | 0 | 44.44% | 4 | 2.5 |
| Q1: I have good knowledge | 0.00% | 0 | 100.00% | 2 | 0.00% | 0 | 0.00% | 0 | 22.22% | 2 | 3 |
| Q1: I have some knowledge | 0.00% | 0 | 0.00% | 0 | 0.00% | 0 | 0.00% | 0 | 0.00% | 0 | 0 |
| Total | 11.11% | 1 | 22.22% | 2 | 33.33% | 3 | 0.00% | 0 | 100.00% | 9 | |

**Answered** 9

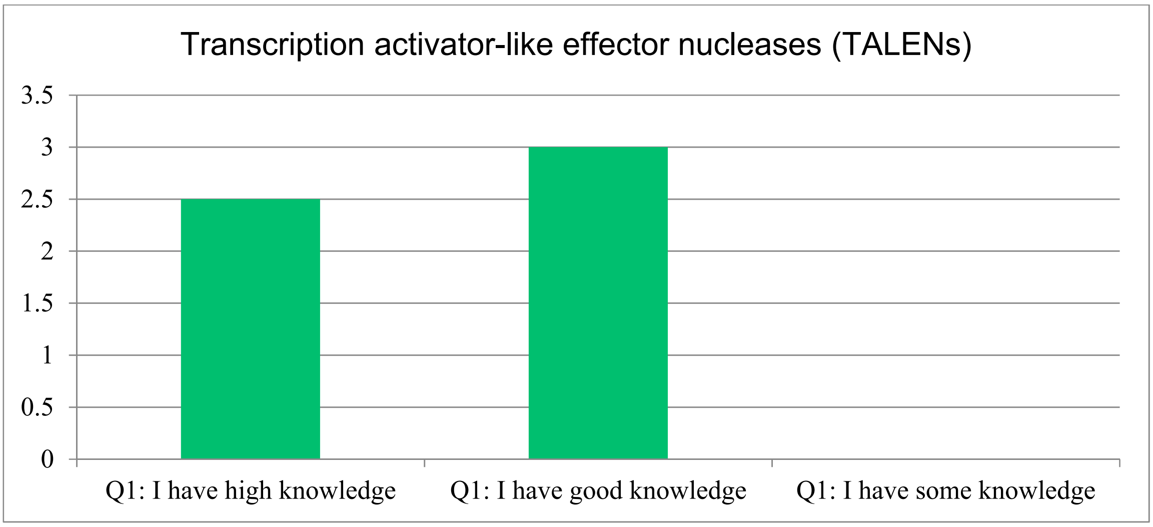


## Meganucleases (MNs)

| | 1 | | 2 | | 3 | | 4 | | Total | | Score |
|---|---|---|---|---|---|---|---|---|---|---|---|
| Q1: I have high knowledge | 0.00% | 0 | 25.00% | 1 | 0.00% | 0 | 75.00% | 3 | 44.44% | 4 | 1.5 |
| Q1: I have good knowledge | 0.00% | 0 | 0.00% | 0 | 100.00% | 2 | 0.00% | 0 | 22.22% | 2 | 2 |
| Q1: I have some knowledge | 0.00% | 0 | 0.00% | 0 | 0.00% | 0 | 0.00% | 0 | 0.00% | 0 | 0 |
| Total | 0.00% | 0 | 11.11% | 1 | 22.22% | 2 | 33.33% | 3 | 100.00% | 9 | |

**Answered** 9

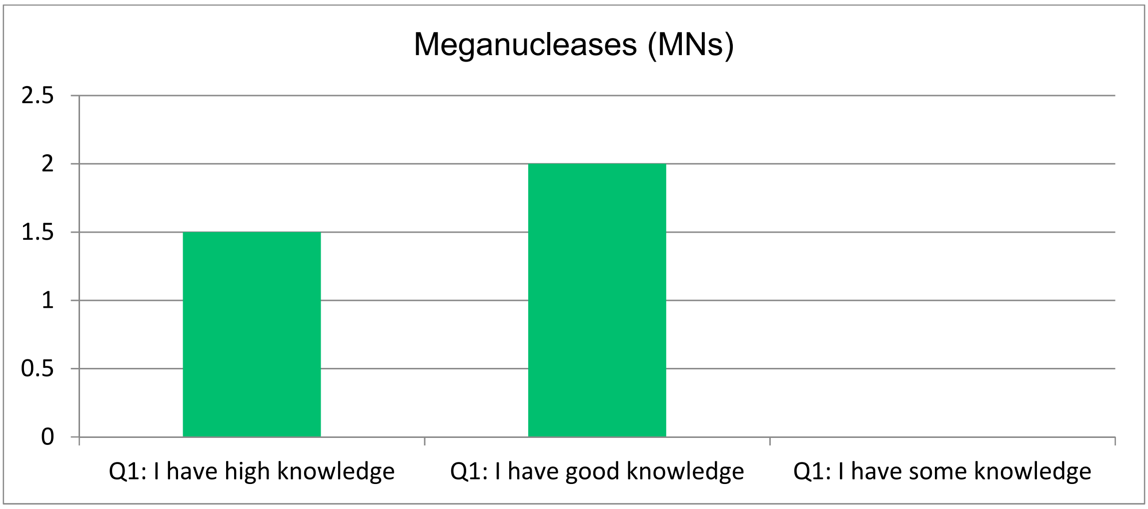


## Zinc finger nucleases (ZFNs)

| | 1 | | 2 | | 3 | | 4 | | Total | | Score |
|---|---|---|---|---|---|---|---|---|---|---|---|
| Q1: I have high knowledge | 0.00% | 0 | 75.00% | 3 | 0.00% | 0 | 25.00% | 1 | 44.44% | 4 | 2.5 |
| Q1: I have good knowledge | 100.00% | 2 | 0.00% | 0 | 0.00% | 0 | 0.00% | 0 | 22.22% | 2 | 4 |
| Q1: I have some knowledge | 0.00% | 0 | 100.00% | 1 | 0.00% | 0 | 0.00% | 0 | 11.11% | 1 | 3 |
| Total | 22.22% | 2 | 44.44% | 4 | 0.00% | 0 | 11.11% | 1 | 100.00% | 9 | |
| | | | | | | | | | | **Answered** | **9** |

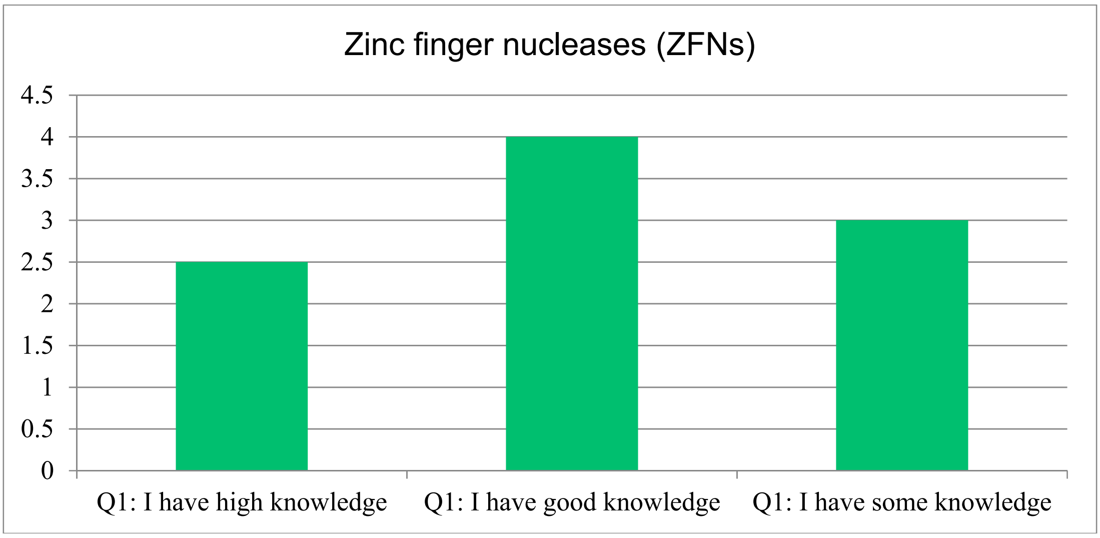

Supplement: Supplementary file 1 [file jcm-11-01283-s001.zip › jcm-1541314-supplementary.pdf]
